# In *silico* evidence implicating novel mechanisms of *Prunella vulgaris* L*.* as a potential botanical drug against COVID-19-associated acute kidney injury

**DOI:** 10.3389/fphar.2023.1188086

**Published:** 2023-05-18

**Authors:** Xue-Ling Yang, Chun-Xuan Wang, Jia-Xing Wang, Shi-Min Wu, Qing Yong, Ke Li, Ju-Rong Yang

**Affiliations:** ^1^ Department of Nephrology, The Third Affiliated Hospital of Chongqing Medical University, Chongqing, China; ^2^ Core Research Laboratory, The Second Affiliated Hospital, Xi’an Jiaotong University, Xi’an, China; ^3^ Beijing Key Laboratory of Bioprocess, College of Life Science and Technology, Beijing University of Chemical Technology, Beijing, China

**Keywords:** COVID-19, acute kidney injury, cytokine storm, network pharmacology, molecular docking

## Abstract

COVID-19-associated acute kidney injury (COVID-19 AKI) is an independent risk factor for in-hospital mortality and has the potential to progress to chronic kidney disease. *Prunella vulgaris* L*.,* a traditional Chinese herb that has been used for the treatment of a variety of kidney diseases for centuries, could have the potential to treat this complication. In this study, we studied the potential protective role of *Prunella vulgaris* in COVID-19 AKI and explored its specific mechanisms applied by network pharmacology and bioinformatics methods. The combination of the protein-protein interaction network and Gene Ontology and Kyoto Encyclopedia of Genes and Genomes enrichment -target gene network revealed eight key target genes (VEGFA, ICAM1, IL6, CXCL8, IL1B, CCL2, IL10 and RELA). Molecular docking showed that all these eight gene-encoded proteins could be effectively bound to three major active compounds (quercetin, luteolin and kaempferol), thus becoming potential therapeutic targets. Molecular dynamics simulation also supports the binding stability of RELA-encoded protein with quercetin and luteolin. Together, our data suggest that IL6, VEGFA, and RELA could be the potential drug targets by inhibiting the NF-κB signaling pathway. Our *in silico* studies shed new insights into *P. vulgaris* and its ingredients, e.g., quercetin, as potential botanical drugs against COVID-19 AKI, and warrant further studies on efficacy and mechanisms.

## 1 Introduction

Coronavirus disease 2019 (COVID-19) is an extremely contagious disease caused by the severe acute respiratory syndrome coronavirus 2 (SARS-CoV-2) that emerged suddenly in 2019 (Hu et al., 2021a). The World Health Organization (WHO) has reported 632 million confirmed cases and 6.5 million deaths as of 13 November 2022 (Dong et al., 2020). The continuous outbreak of COVID-19 poses a great threat not only to human health but also to the global economy, society and human life (Daniels and Morton, 2023). Although a large number of studies have been conducted focusing on the prevention and treatment of the disease, the mechanism behind treatment efficacy is not fully understood.

The main symptoms of COVID-19 are fever, cough and dyspnoea, and some patients in the acute phase may experience acute respiratory distress syndrome, sepsis, acute cardiac injury and secondary infection. Moreover, patients may develop multiple organ failure and shock in severe cases (Huang et al., 2020a). For instance, studies have reported a relatively high incidence rate of acute kidney injury (AKI, up to 77%) in patients with COVID-19, especially among those in the ICU (Huang et al., 2020b; Chen et al., 2020; Cheng et al., 2020; Guan et al., 2020; Yang et al., 2020; Matsumoto and Prowle, 2022). The chief pathologic change of AKI is renal tubular damage, with major physical signs of hematuria and proteinuria (Kudose et al., 2020; Han and Ye, 2021; Lin et al., 2022). Studies have reported that COVID-19-associated AKI (COVID-19 AKI) (Nadim et al., 2020) is not only linked to high in-hospital mortality rates but also accompanied by long-term complications, such as persistent AKI, end-stage renal disease and chronic renal insufficiency (Lin et al., 2020; Matsumoto and Prowle, 2022). Therefore, attention must be paid to the occurrence of AKI in patients with COVID-19, and early prevention, timely diagnosis and monitoring are important.

There is limited knowledge about the pathogenesis of AKI related to SARS-CoV-2; however, the current mainstream views are as follows. First, SARS-CoV-2 binds to ACE2 receptors on the surface of resident renal cells, including podocytes and tubular cells, to enter the cells and thus exert a direct damaging effect. Second, SARS-CoV-2 N protein can interact with Smad3 to form a complex to promote Smad3 signaling in response to TGF-β1and activate Smad3 to induce kidney cell death and cause acute kidney injury (AKI) (Wang et al., 2022). Third, the virus-induced cytokine storm and inflammation cause damage to the kidney (Ahmadian et al., 2021). Fourth, vascular endothelial dysfunction, coagulation dysfunction and complement activation may also be important mechanisms for the development of AKI in a subset of patients with COVID-19 (Nadim et al., 2020; Ahmadian et al., 2021; Han and Ye, 2021).

The efficacy of traditional Chinese medicine (TCM) in prevention and control of COVID-19, with medications having antiviral, anti-inflammatory and immune regulatory properties, can significantly reduce the hospitalisation rate and shorten the symptom recovery time (Runfeng et al., 2020; Hu et al., 2021b; Gajewski et al., 2021). *Prunella vulgaris* L*.* is a Chinese herbal medicine effective against a variety of diseases and has significant antiviral, anti-inflammatory, anti-oxidative and immunomodulatory effects (Bai et al., 2016). Early studies have reported inhibition of *Prunella vulgaris* on HIV, HSV and Ebola infection (Tabba et al., 1989; Yao et al., 1992; Xu et al., 1999; Zhang et al., 2016). In addition, APV can exert a significant antiviral effect by blocking the entry of SARS-CoV-2 into cells (Ao et al., 2021). What is more, *P. vulgaris* is the principal ingredient of Xia Sang Ju formula, a 3-herb traditional herbal remedy recommended for COVID-19 treatment by the Chinese government, and it alone can also be used as an herbal tea. APV can also protect the kidney through its anti-inflammatory and anti-oxidative effects, which are accomplished by effectively inhibiting the activation, translocation of nuclear factor kappa-B (NF-kB), and generation of ROS, thereby inhibiting the expression of inflammatory factors, such as intracellular cell adhesion molecule-1 (ICAM-1) and monocyte chemoattractant protein-1 (MCP-1) (Namgung et al., 2017). These findings suggest a potential therapeutic role for *P. vulgaris* in COVID-19 AKI; however, the exact mechanism has not been fully investigated.

Network pharmacology is a method for the systematic construction of the ingredients-target-disease network. It reveals the potential pathogenetic molecular mechanisms in a high-throughput manner and provides a new scientific paradigm for the study of Chinese medicine (Nogales et al., 2022). In this study, we aim to gain novel mechanistic insights into *P. vulgaris* and its ingredients, as potential botanical drugs against COVID-19 AKI and to guide further translational research and development. Our research provides new therapeutic ideas and targets for COVID-19 AKI.

## 2 Materials and methods

### 2.1 Obtaining active pharmaceutical ingredients and protein targets

The Traditional Chinese Medicine Systems Pharmacology Database (TCMSP, http://tcmspw.com) was used to search for the main ingredients of *P. vulgaris*, following which the active molecules were filtered by setting a pharmacokinetic index, which considers oral bioavailability (OB) greater than 30% and drug-like (DL) index >0.18 (Ru et al., 2014; Zheng and Wang, 2014). The targets of all active ingredients were downloaded, and protein IDs, gene symbols, as well as annotations were obtained using the Uniprot protein database (https://www.uniprot.org) and STRING database (https://string-db.org) (Consortium, 2015).

### 2.2 Prediction of disease targets

The COVID-19-related target genes were downloaded from the GeneCards database (http://www.genecards.org), Treatment Target Database (TTD, https://db.idrblab.org/ttd) and Human Online Mendelian Inheritance database (OMIM, https://OMIM.org) (Rebhan et al., 1997; Chen et al., 2002; Amberger et al., 2015). The AKI-related target genes were then downloaded from the DisGeNET database (http://www.disgenet.org) and the GeneCards database (Piñero et al., 2017). The intersection target of COVID-19 and AKI was obtained with the Wayne analysis tool, and these target genes were considered the relevant genes for COVID-19-induced AKI. The disease target genes were then intersected with the drug target genes to obtain the drug-disease intersection genes and were considered the possible target genes for *P. vulgaris* against COVID-19 AKI. The Venn diagram was drawn using the online Venny platform (https://bioinfogp.cnb.csic.es/tools/Venny/index.html).

### 2.3 Construction of the compound-target gene network

We used Cytoscape 3.9.1 (http://www.Cytoscape.org) to construct a network of compound-target interactions between the aforementioned drug-disease intersection targets and active compounds of the drug (Shannon et al., 2003). In the network, nodes represent the selected active compounds and targets, while the edges between the nodes represent the interactions between these molecules and genes. The connectivity between molecules and targets in the core structure of the network was determined by means of betweenness (implemented by the plug-in CytoNCA), and the molecule with a larger value was more likely to become a critical compound of COVID-19 AKI (Xia et al., 2020).

### 2.4 Construction of the protein-protein interaction network

The aforementioned disease-drug intersection gene targets were imported into the STRING database (https://string-db.org) to construct the protein-protein interaction (PPI) network (Szklarczyk et al., 2019). Species were selected as *Homo sapiens*, the meaning of network interaction was set as evidence, active sources involved Textmining, Experiments, Databases, Co-expression, Neighborhood, Gene Fusion and Co-occurrence and the minimum interaction score was set as medium confidence (0.4). Following this, the PPI network results of STRING were imported into Cytoscape 3.9.1, and the CytoNCA plugin was used to analyse network topology characteristics, with betweenness as an index to select key target genes by which *P. vulgaris* treat COVID-19 AKI.

### 2.5 GO functional enrichment and KEGG pathway analysis

Gene ontology (GO) functional annotation and Kyoto Encyclopedia of Genes and Genomes (KEGG) pathway enrichment analyses were performed for the aforementioned disease-drug intersection gene targets to reveal potential mechanisms based on biological processes (BP), cellular compounds (CC), molecular functions (MF), and critical signaling pathways. These were implemented using the ClusterProfiler package in R (version 4.1.2) (Li et al., 2021). *p*-values <0.05 and q-value (an adjusted *p*-value, taking in to account the false discovery rate) < 0.05 were considered statistically significant. In addition, visual analysis was performed using the ggplot package and pathview package in R. Cytoscape was used to network all the top 15 enriched BP, CC, MF and the top 20 KEGG pathways that intersected with the aforementioned disease-drug gene targets to identify the genes that appeared most in these enriched results.

### 2.6 Molecular docking technology

First, we downloaded and processed the protein structures for molecular docking. The three-dimensional structures of the proteins were downloaded from the RCSB Protein Data Bank (http://www.rcsb.org) (Berman et al., 2000). Crystal structures of proteins of human origin with a resolution greater than 2 Å were selected, and pdb files with high resolution and single proteins were downloaded. The proteins were processed using AutoDock Tools (version 1.5.6) (Morris et al., 2009), including water removal, hydrogenation, charge calculation and addition of atom type. Finally, the processed proteins were saved as PDBQT files. We then downloaded and processed the ligands for molecular docking. The 3D structure of the molecule was downloaded from PubChem (https://pubchem.ncbi.nlm.nih.gov/) and processed using AutoDock Tools (version 1.5.6) (Wang et al., 2017), involving adjusting charge, detecting and selecting rotatable keys of ligands. Molecular docking was performed using AutoDock Vina 1.12 with the setting of exhaustiveness = 10 and num_modes = 10. Each docking calculation generates ten structures that construct the least energetic ligand-protein complexes. Finally, 3D structure visualisation and 2D interaction mapping of proteins and ligands were performed using Discovery Studio Visualizer 2019, and the docking results were visualised using PyMol 2.5.2.

### 2.7 Molecular dynamics (MD)simulation

MD simulation were performed using Amber 22 (San Francisco, CA, United States of America) (Case et al., 2022). The ff19SB force field (Tian et al., 2019) was used to calculate the system force field parameters. Solvation was performed using the TIP3P water model, and Na^+^ and Cl^−^ were added to neutralize the system. Once the system energy was minimised, the system was heated from 0 K to 300 K within 500 ps. System confinement was performed in a canonical ensemble system synthesis, followed by system pre-equilibration at 300 K. Finally, 200 ns MD simulations were carried out in an isothermal isobaric system synthesis, maintaining periodic boundary conditions. All covalent bonds involving hydrogen were constrained by the SHAKE method. The root mean square deviation (RMSD), the root mean square value of atomic fluctuations (RMSF), Solvent accessible surface area (SASA) (Weiser et al., 1999), the free energy of the binding reaction and hydrogen binding were analysed. RMSD and RMSF were defined as 1) and 2), respectively.

$$\begin{array}{c} RMSD=\sqrt{\frac{\sum_{n=1}^{N}\left[m_{i}*{\left(X_{i}-Y_{i}\right)}^{2}\right]}{M}} \end{array}$$ (1)

$$\begin{array}{c} RMSF=\sqrt{\frac{\sum_{t_{j}=1}^{T}{\left|X_{i}\left(t_{j}\right)-Y_{i}\right|}^{2}}{M}} \end{array}$$ (2)

## 3 Results

### 3.1 Acquisition of the P. vulgaris-target and COVID-19 AKI-associated genes

**FIGURE 1:**
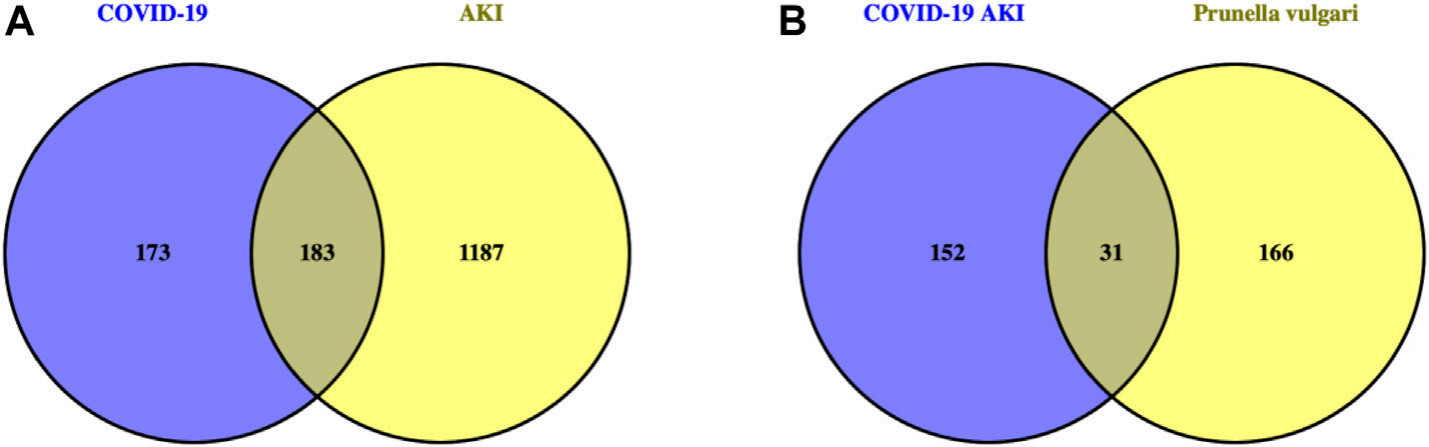
Venn diagram identifies intersection targets of drug and disease. **(A)**. The intersection genes of AKI with COVID-19 are potential targets for COVID-19 induced AKI. **(B)**. The intersection targets of *Prunella vulgaris* with COVID-19 AKI are possible target genes for *Prunella vulgaris* against COVID-19 AKI.

We obtained 11 active compounds of *P. vulgaris* from the TCMSP database (Table 1), and a total of 197 drug target genes were obtained after de-duplication. In addition, we obtained 372, 52 and three genes related to COVID-19 from the GeneCards, OMIM and TTD databases, respectively, and finally acquired 356 targets after de-duplication. Following this, 163 and 1,315 AKI-related target genes were obtained from the DisGeNET and GeneCards databases, respectively, and finally 1,370 genes were obtained after de-duplication. The intersection of the target genes of AKI and COVID-19 yielded 183 genes that can be considered potential target genes for COVID-19 AKI (Figure 1A).

**TABLE 1:** Active ingredients of *Prunella vulgaris.*

Mol ID^a^	Molecule name	OB^b^ (%)	DL^c^
MOL000006	luteolin	36.16	0.25
MOL000098	quercetin	46.43	0.28
MOL000358	beta-sitosterol	36.91	0.75
MOL000422	kaempferol	41.88	0.24
MOL000449	stigmasterol	43.83	0.76
MOL000737	morin	46.23	0.27
MOL004355	spinasterol	42.98	0.76
MOL004798	delphinidin	40.63	0.28
MOL006767	vulgaxanthin-I	56.14	0.26
MOL006772	poriferasterol monoglucoside_qt	43.83	0.76
MOL006774	stigmast-7-enol	37.42	0.75

^a^ Mol ID, indicates the ID, of the drug compound defined in the TCMSP.

^b^ OB (Oral Bioavailability) is defined as “the rate and extent to which the active ingredient or active moiety is absorbed from a drug product and becomes available at the site of action”.

^c^ DL (Drug-likeness) index represents the similarity between the composition and known chemical medicine.

### 3.2 Construction of the active compound-target network

**FIGURE 2:**
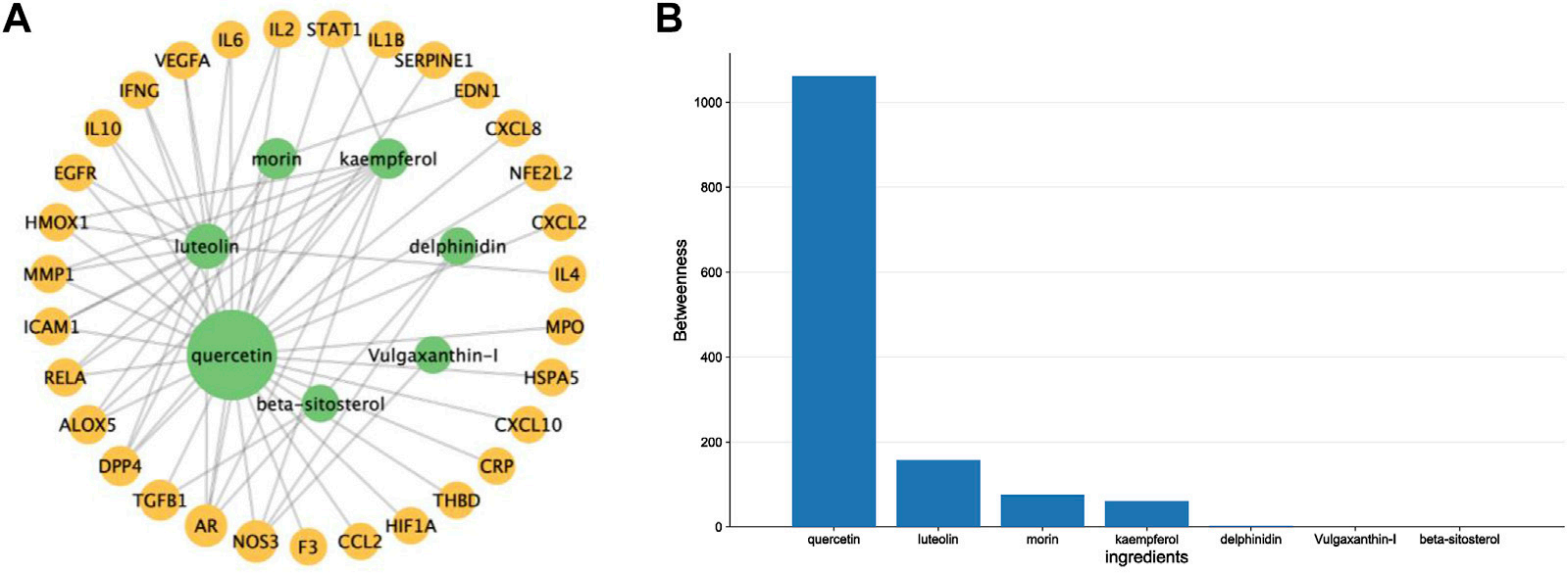
Constructing a drug-ingredients-target network to identify the crucial compounds. **(A)**. A network diagram of *Prunella vulgaris* compound-target interactions was constructed by Cytoscape 3.9.1, and node size indicated the value of betweenness. **(B)**. Histograms show betweenness for the ingredients, and the molecule with a larger value was more likely to become a critical compound of COVID-19 AKI.

By intersecting drug target genes and disease-related genes, we finally obtained a relevant gene set, comprising 31 genes, for *P. vulgaris* treating COVID-19 AKI (Figure 1B). Based on the targets of the active ingredient, we found that this intersection gene set was associated with only seven active ingredients (quercetin, luteolin, morin, kaempferol, delphinidin, vulgaxanthin-I and beta-sitosterol). A composite active compounds-target interaction network with 38 nodes (31 genes and seven compounds) and 59 edges (Figure 2A) was visualised using Cytoscape 3.8.0, and node size indicated the importance of the node. We plotted the histogram of the seven chemical compounds based on the results of betweenness (indicates the proportion of the shortest paths through node to all the shortest paths) (Figure 2B) and found that five compounds, including quercetin, luteolin, morin, kaempferol and delphinidin, were more important.

### 3.3 Construction of the PPI network

**FIGURE 3:**
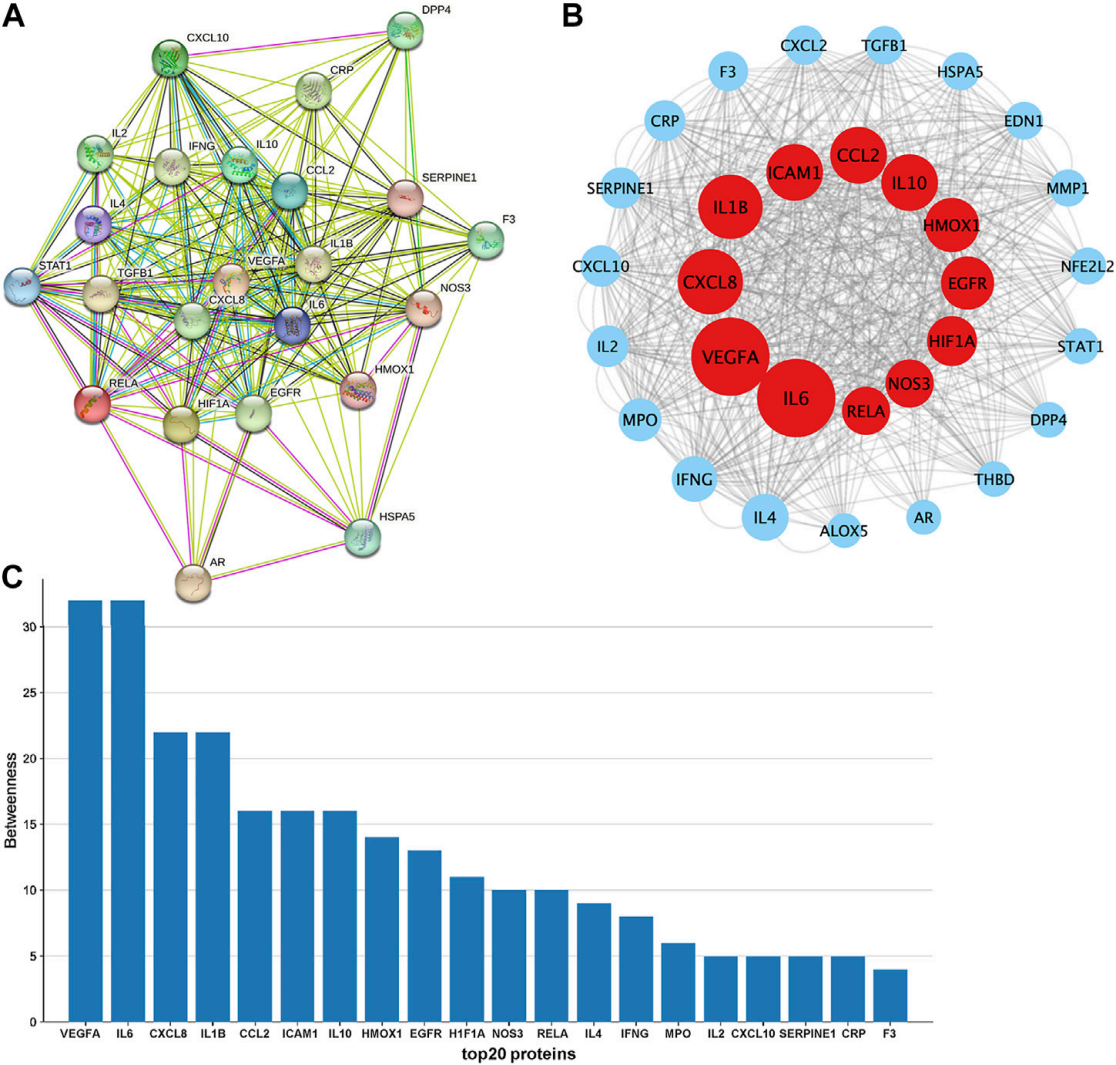
Constructing a PPI network to find essential target genes. **(A)**. PPI network map of targets was downloaded from the STRING database. **(B)**. PPI network map was further visualized with Cytoscape software. **(C)**. Target genes representing the top 20 of betweenness were visualised with histograms.

The PPI (Figure 3A) network of the 31 drug-disease intersection genes was exported from the STRING database, and the result showed that the proteins encoded by these target genes had complex interactions. We imported the PPI network into Cytoscape for further analysis. The CytoNca plugin was used to rank the strength of action of these proteins according to the betweenness, and each node showed different circle sizes, with larger circles having higher values (Figure 3B). The top 20 ranked genes were visualised on a histogram (Figure 3C). The top 10 genes with the strongest protein interactions were IL6, VEGFA, CXCL8, IL1B, ICAM1, CCL2, IL10, HMOX1, EGFR and HIF1A.

### 3.4 GO enrichment analysis

**FIGURE 4:**
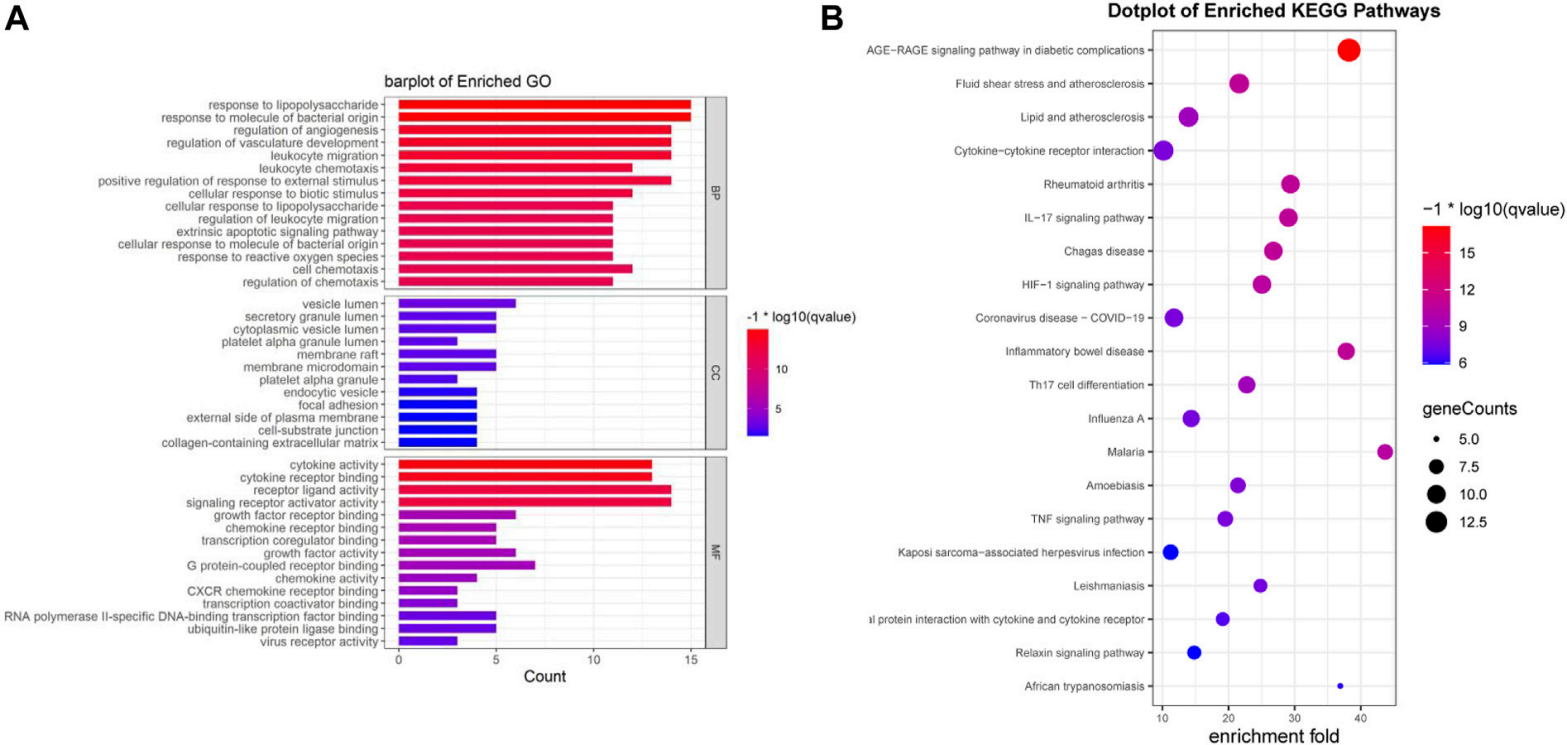
Enrichment analysis was performed for intersecting genes. **(A)**. A total of top 15 BP, CC and MF terms are visualised with bar chat. **(B)**. Top 20 enriched KEGG terms are visualised with bubble chart. (*p-value<0.05, *q-value<0.05).

We performed an enrichment analysis of GO functional annotation to understand the function of intersecting genes. A total of 1,560 GO terms, including 1,510, 12 and 38 biological processes (BP), cellular compounds (CC), molecular functions (MF), respectively, and the top 15 terms were identified (Figure 4A). BP are mainly enriched in cells and the body in response to external stimuli and bacteria, inflammatory responses (leukocyte migration and chemotaxis), regulation of reactive oxygen species (ROS) and apoptosis, in addition to the regulation of angiogenesis and vascular development. CC are mainly concentrated in cell membranes, vesicles, secretory granules and the extracellular matrix. MF is mainly manifested in cytokines, receptor ligands, chemokines, growth factor activity and viral receptor activity. We have presented the results of all 10 GO terms associated with viral invasion after analysis (Table 2), which showed mechanisms that could inhibit SARS-CoV-2 mainly as follows: response to virus, defense response to virus, viral life cycle, cellular response to virus, viral entry into host cell.

**TABLE 2:** Thirteen virus-related GO enrichment terms.

Ontology	ID	Description	Enrichment fold^a^	q-value^b^	Count
BP	GO:0009615	response to virus	11.52	5.69E-06	7
BP	GO:0016032	viral process	10.19	1.11E-05	7
BP	GO:0051607	defense response to virus	13.67	1.11E-05	6
BP	GO:0019058	viral life cycle	9.53	0.00023579	5
BP	GO:0098586	cellular response to virus	21.57	0.00045209	3
BP	GO:0046718	viral entry into host cell	12.58	0.00156,767	3
BP	GO:0050688	regulation of defense response to virus	17.51	0.00434,012	2
BP	GO:0019079	viral genome replication	9.22	0.01,163,095	2
BP	GO:0044793	negative regulation by host of viral process	40.26	0.01,342,401	1
BP	GO:0140374	antiviral innate immune response	40.26	0.01,342,401	1
MF	GO:0001618	virus receptor activity	23.39	0.00142,444	3
BP	GO:0002230	positive regulation of defense response to virus by host	38.97	0.0011587	2
BP	GO:0050691	regulation of defense response to virus by host	29.46	0.001846	2

^a^ Enrichment fold means a value of the percentage of genes in list belonging to the pathway, divided by the corresponding percentage in the background.

^b^ q-value represents an adjusted *p*-value, taking in to account the false discovery rate.

### 3.5 KEGG pathway analysis

**FIGURE 5:**
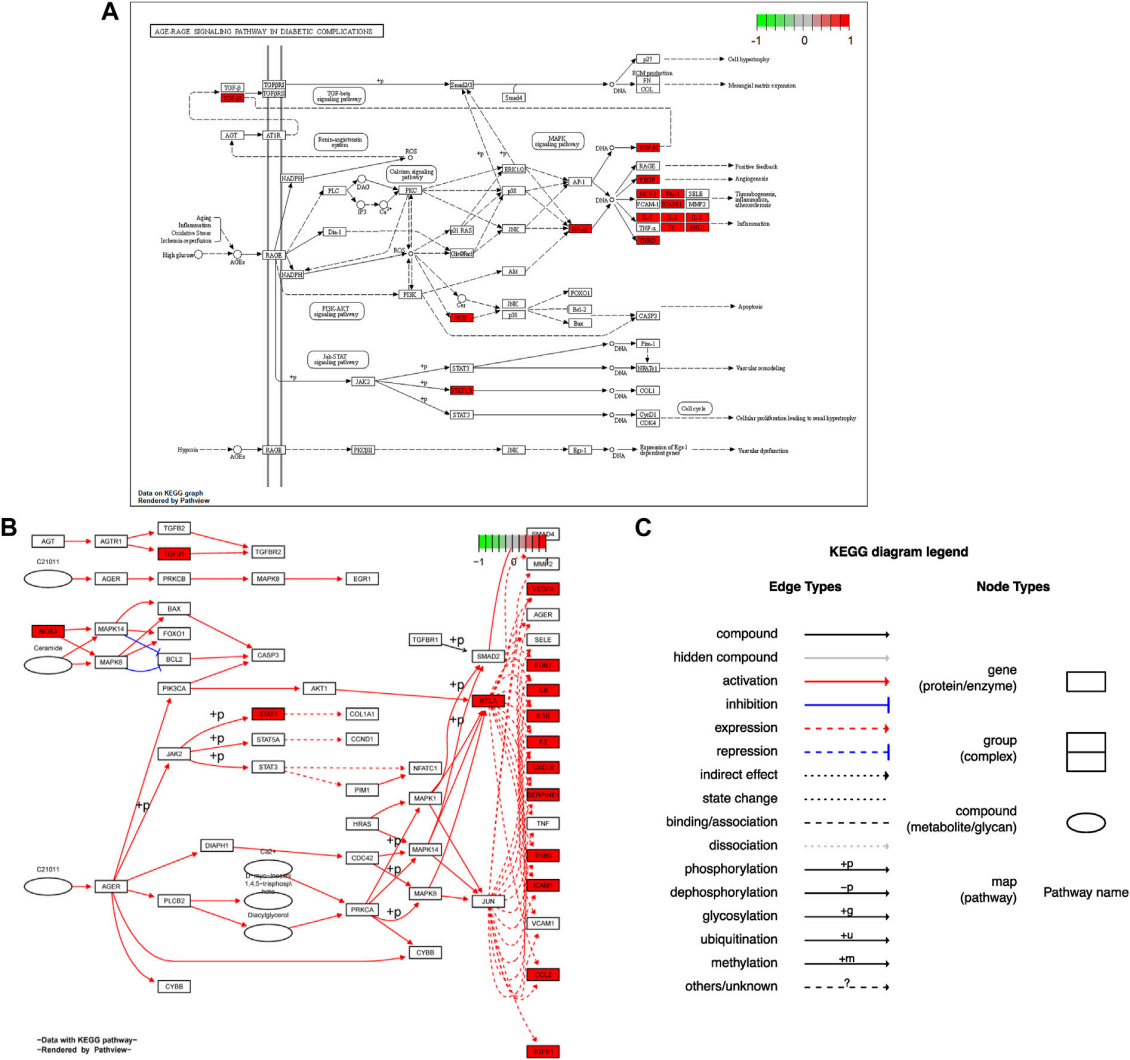
Hsa 04933 KEGG pathway map were acquired from pathview package in R. **(A)**. Protein KEGG pathway map was present. **(B)**. Gene KEGG pathway map was shown. **(C)**. Annotation of gene KEGG pathway map was obtained.

**FIGURE 6:**
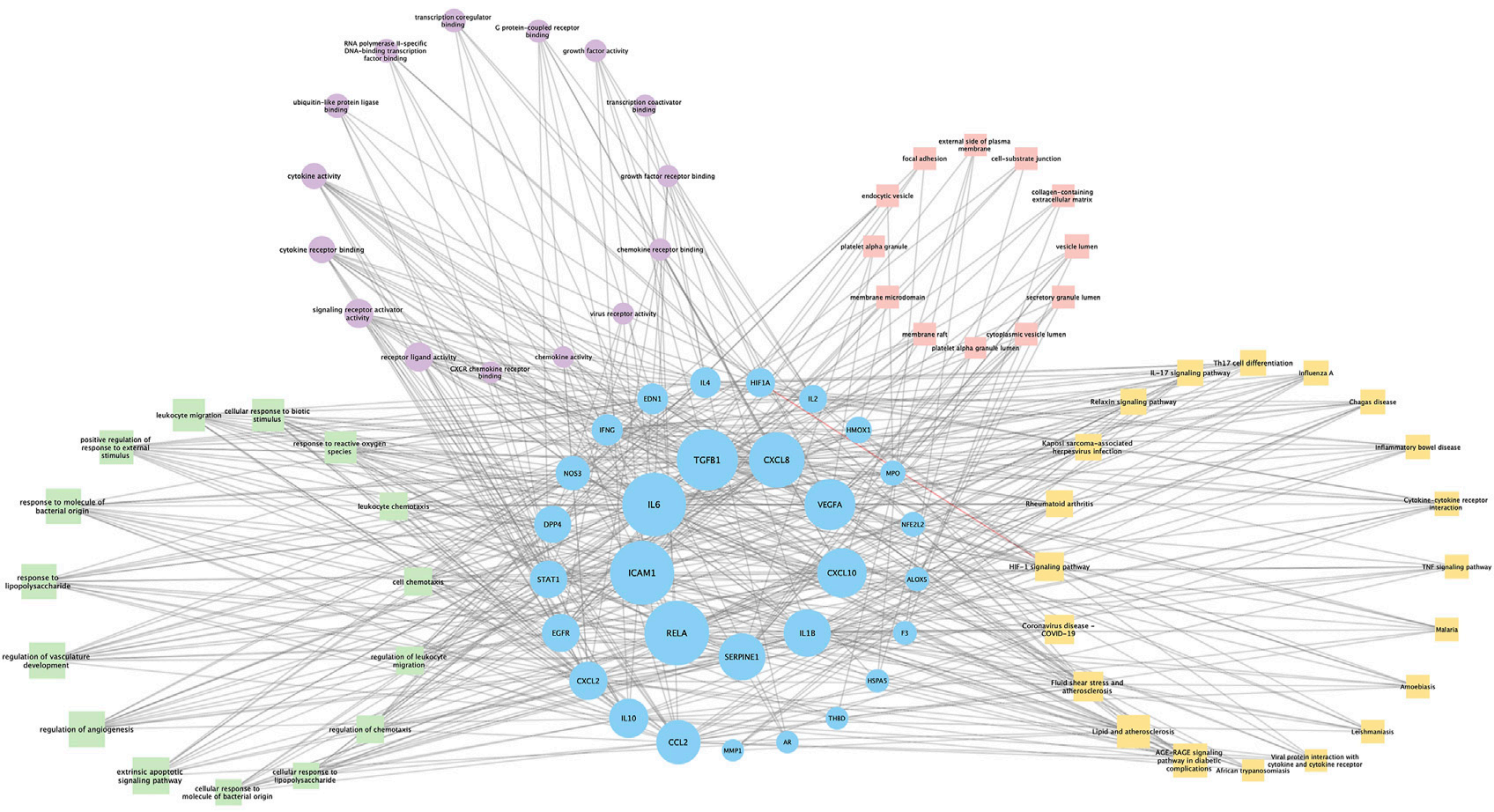
Constructing a GO and KEGG-target interaction network to find essential target genes.

Seventy-six terms were enriched through the KEGG pathway enrichment analysis, ranked by q-value, and the top 20 KEGG terms were identified (Figure 4B). The KEGG suggests that these target genes are associated with a series of important pathological processes, such as the route of infection by external microorganisms (bacteria, viruses and protozoa), the differentiation of immune cells, and inflammation-related pathways (signal transduction pathways). We have presented the top enriched pathway map, the advanced glycation end products (AGEs)- regulation of the receptor for AGEs (RAGE) signaling pathway in diabetic complications, which shows that *P. vulgaris* may regulate VEGFA, IL6, IL-8 and ICAM1 expression through RELA (Figure 5A), thereby alleviating angiogenesis, thrombosis and the inflammatory response (Figures 5B,C). All top 20 KEGG pathway maps have been provided (Supplementary File S1). In addition, we have presented 13 terms associated with viral invasion based on the results of the KEGG analysis (Table 3), which suggests that these target genes play an essential role in viral infection. The top 15 BP, 12 CC, 15 MF, and the top 20 KEGG pathways and their associated genes were visualised using Cytoscape (Figure 6). The top nine genes enriched were RELA, ICAM1, IL6, TGFB1, CXCL8, VEGFA, CXCL10, IL1B and SERPINE1. Furthermore, in combination with the results of the PPI network analysis, we identified eight key target genes of *P. vulgaris* associated with COVID-19 AKI, including IL6, VEGFA, CXCL8, IL1B, ICAM1, CCL2, IL10 and RELA.

**TABLE 3:** Ten virus-associated KEGG pathways.

ID	Description	Enrichment fold^a^	q-value^b^	Count
hsa05171	Coronavirus disease - COVID-19	11.77	2.90E-08	10
hsa05164	Influenza A	14.37	3.24E-08	9
hsa04061	Viral protein interaction with cytokine and cytokine receptor	19.11	2.52E-07	7
hsa05167	Kaposi sarcoma-associated herpesvirus infection	11.26	1.30E-06	8
hsa05163	Human cytomegalovirus infection	8.49	3.14E-05	7
hsa05162	Measles	9.82	0.00023886	5
hsa05160	Hepatitis C	8.7	0.00037313	5
hsa05161	Hepatitis B	8.43	0.00042346	5
hsa05169	Epstein-Barr virus infection	6.76	0.00112,339	5
hsa05166	Human T-cell leukemia virus 1 infection	6.15	0.00165,752	5

^a^ Enrichment fold means a value of the percentage of genes in list belonging to the pathway, divided by the corresponding percentage in the background.

^b^ q-value represents an adjusted *p*-value, taking in to account the false discovery rate.

### 3.6 Molecular docking of active compounds and eight key proteins

**FIGURE 7:**
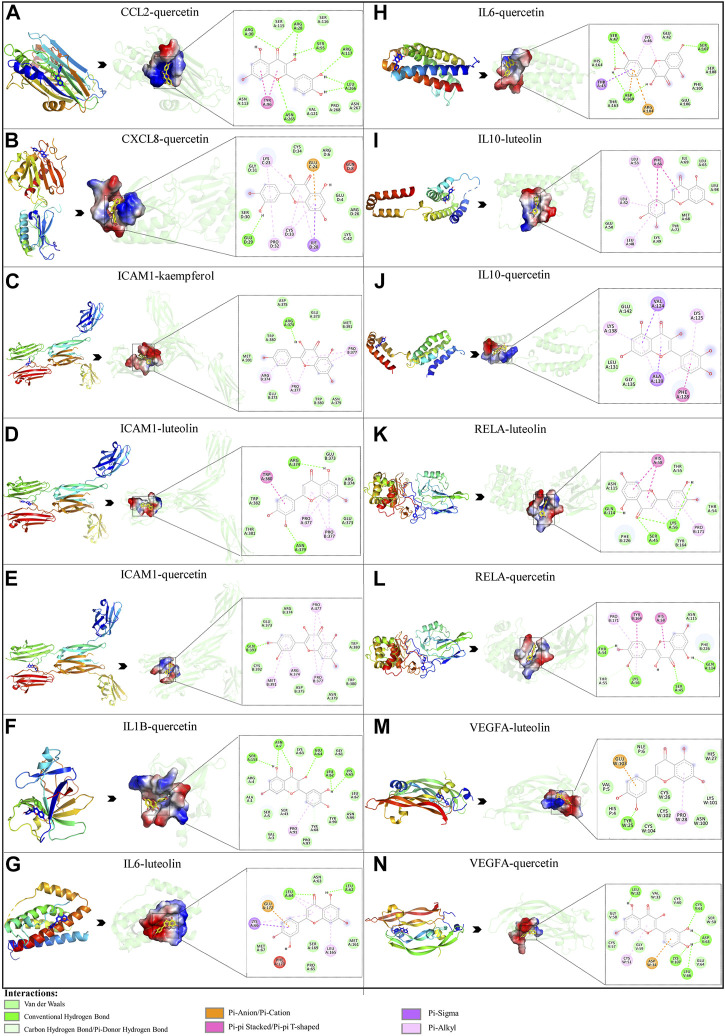
Molecular docking of three active compounds and eight proteins were performed. **(A**–**N)** A total of 14 docking results were obtained. On the left of each docking result, 3D structures of ligands and receptors are shown, with active pockets of proteins highlighted in the middle and 2D interaction maps on the right.

Based on the previous results, we identified eight key proteins that play critical roles in the effects of *P. vulgaris* on COVID-19 AKI and obtained their 3D structures from the RCSB Protein Data Bank (Table 4). Based on the identified drug compound targets, we found that only three drug molecules (quercetin, luteolin and kaempferol) acted on the aforementioned eight proteins. Table 5 lists these three compounds and their corresponding predicted targets. Quercetin, luteolin and kaempferol were selected for molecular docking, and their 3D structures were downloaded from PubChem (Table 6). As previously described, binding affinity less than −5.0 and −7.0 kcal/mol suggests good and strong binding activities, respectively (Liu et al., 2022). The molecular docking results (Figure 7) revealed that the three predicted ingredients demonstrated a good docking effect with their corresponding targets, and the interactions were relatively strong. Quercetin had a good affinity for all eight key proteins, luteolin had a good binding ability for RELA, ICAM1, VEGFA, IL6 and CXCL8, and kaempferol could effectively bind to ICAM1. Thus, the results suggested that these three active compounds, especially quercetin, exerted protective effects against COVID-19 AKI by regulating the aforementioned key target genes.

**TABLE 4:** Eight candidate protein structures.

Protein	PDB index	Structure without water and ligand
ICAM1	1P53	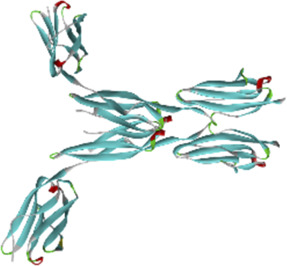
IL8	1QE6	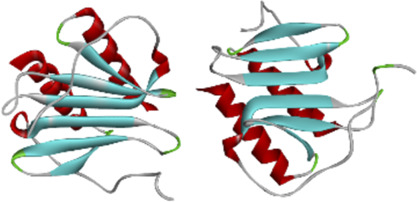
IL1B	1TWM	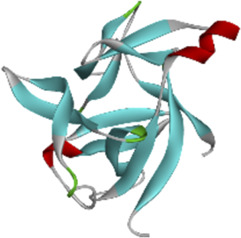
IL10	2H24	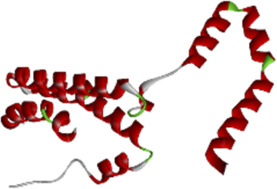
IL6	4NI7	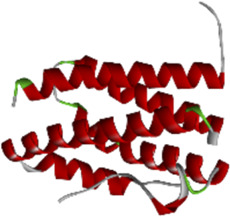
CCL2	4ZK9	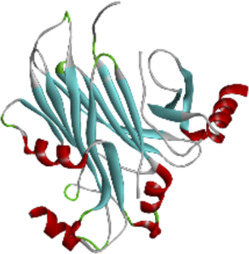
RELA	6GGR	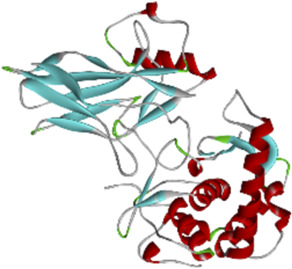
VEGFA	6ZBR	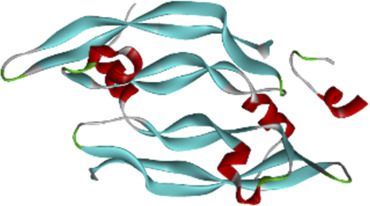

Note: ICAM1, Intercellular Adhesion Molecule 1; IL8, Interleukin 8; IL1B, Interleukin 1 Beta; IL10, Interleukin 10; IL6, Interleukin 6; CCL2, C-C Motif Chemokine Ligand 2; RELA, RELA proto-oncogene (NF-κB subunit); VEGFA, Vascular Endothelial Growth Factor A.

**TABLE 5:** Fourteen kinds of molecular docking results.

Molecule	Protein	Affinity (kcal/mol)
luteolin	RELA	−8.7
quercetin	IL8	−8.4
quercetin	RELA	−7.9
quercetin	ICAM1	−7.3
quercetin	CCL2	−7.3
luteolin	ICAM1	−7.2
quercetin	VEGFA	−7.1
kaempferol	ICAM1	−7
luteolin	VEGFA	−7
quercetin	IL1B	−6.9
quercetin	IL6	−6.9
luteolin	IL6	−6.8
luteolin	IL10	−6.6
quercetin	IL10	−6.5

**TABLE 6:** Molecular information of three candidate ligands.

Molecule name	Compound CID^a^	MF^b^	MW^c^	3D structure
quercetin	5280343	C_15_H_10_O_7_	302.23 g/mol	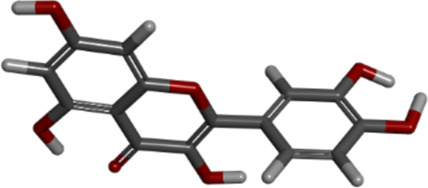
luteolin	5280445	C_15_H_10_O_6_	286.24 g/mol	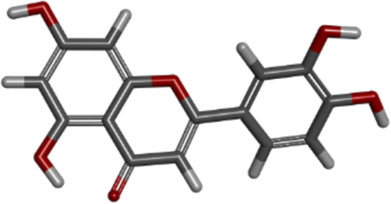
kaempferol	5280863	C_15_H_10_O_6_	286.24 g/mol	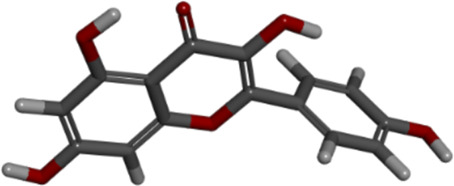

^a^ CID, PubChem Compound Identification.

^b^ MF, molecular formula.

^c^ MW, molecular weight.

### 3.7 MD simulation

**FIGURE 8:**
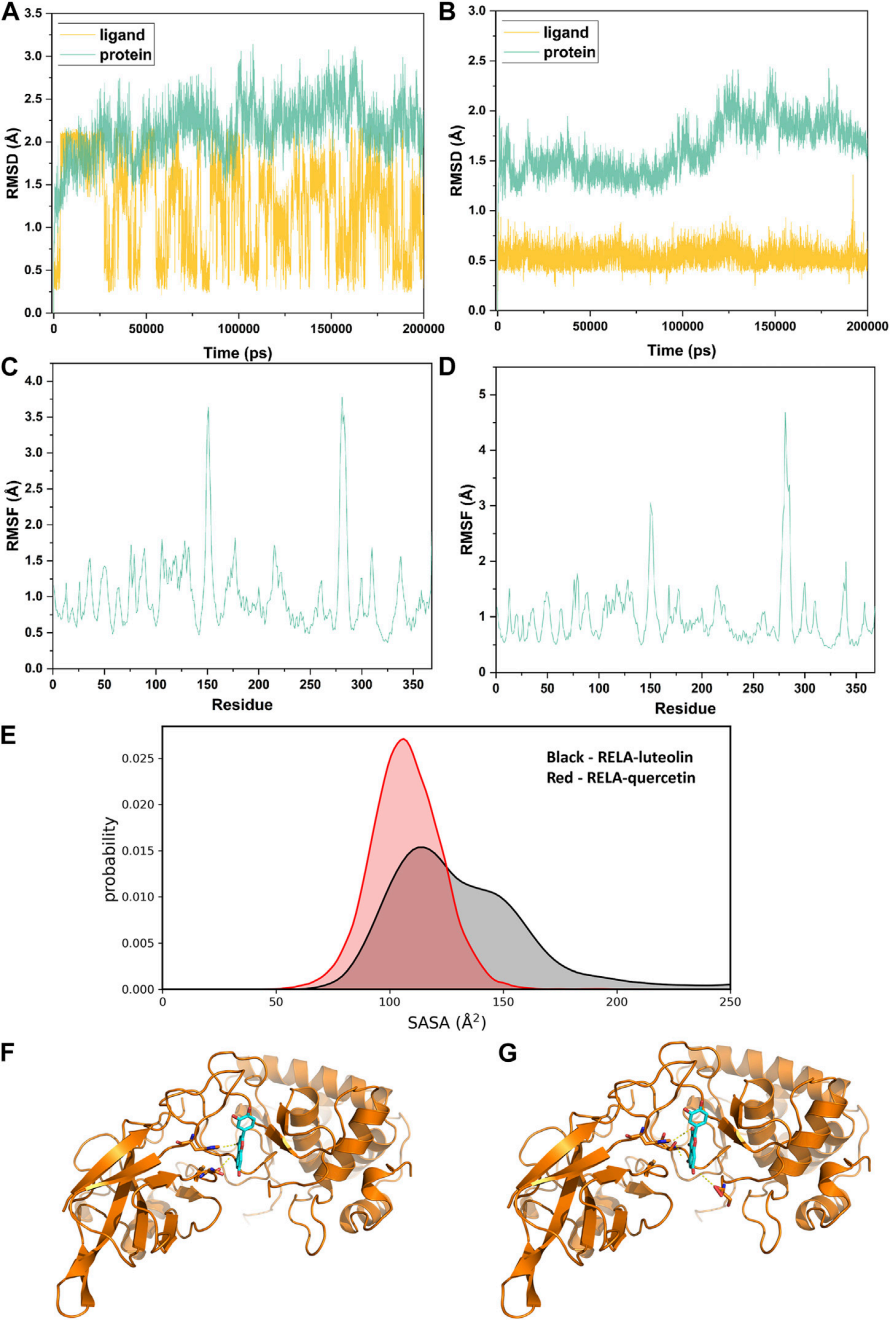
MD simulation analysis. **(A)** and **(B)**. The RMSD value of RELA-luteolin and RELA-quercetin, respectively. **(C)** and **(D)**. Changes in protein flexibility during simulation of luteolin and quercetin, respectively. **(E)**. Relative surface area contribution of the ligand exposed to the solvent of luteolin and quercetin. **(F)** and **(G)**. Kinetic conformation of the RELA-luteolin and RELA-quercetin systems, respectively.

Considering that RELA-encoded protein (NF-κB) presents the highest docking scores with molecules, it was chosen for MD simulations. After 200 ns of long-scale MD simulations, the RMSD trend of protein *α*-carbon atoms (protein backbone) and ligands in the simulated trajectories of the two systems over time showed that the RMSD was small for both RELA-luteolin and RELA-quercetin systems, with the RELA-luteolin system undergoing conformational transitions in a small conformational space and RELA-quercetin system fluctuating in a relatively constant conformational space (Figures 8A,B). The RMSF represents the flexibility changes of the protein during the simulation. The protein in RELA-luteolin and RELA-quercetin shows similar flexibility changes, with larger fluctuations in the loop conformation around residues 150 and 280, consistent with the high flexibility of the loops (Figures 8C,D). The Solvent accessible surface area (SASA) reflects the relative surface area contribution of the ligand exposed to the solvent and the SASA value fluctuated within a relatively constant range for both RELA-luteolin and RELA-quercetin systems (Figure 8E), which is consistent with the stability of the RMSD analysis. Free energy calculations using the Molecular Mechanics-Poisson Bolzmann Surface Area (MM/PBSA) method showed that the free energies of both RELA-luteolin and RELA-quercetin systems were relatively stable (Table 7; Table 8). Additionally, the hydrogen bond analysis indicates strong hydrogen bond interactions in both systems (Figure 8F; Figure 8G; Table 9; Table 10).

**TABLE 7:** MM/PBSA energy analysis of RELA-luteolin.

Energy component	Average	Std. Dev.	Std. Err. Of mean
VDWAALS	−28.039	3.5066	0.4959
EEL	−17.0597	11.6462	1.647
EPB	35.5132	9.9989	1.4141
ENPOLAR	−2.8371	0.1992	0.0282
ΔG	−12.4226	5.6026	0.7923

Note: VDWAALS, van der Waals energy; EEL; electrostatic energy; EPB; energy calculated by Poisson Boltzmann (PB) equation; ENPOLAR; nonpolar energy; ΔG, binding free energy.

**TABLE 8:** MM/PBSA energy analysis of RELA-quercetin.

Energy component	Average	Std. Dev.	Std. Err. Of mean
VDWAALS	−31.235	2.8066	0.3969
EEL	−26.8982	11.6517	1.6478
EPB	46.3668	9.0074	1.2738
ENPOLAR	−3.0681	0.1471	0.0208
ΔG	−14.8345	4.1159	0.5821

Note: VDWAALS, van der Waals energy; EEL, electrostatic energy; EPB, energy calculated by Poisson Boltzmann (PB) equation; ENPOLAR, nonpolar energy; ΔG, binding free energy.

**TABLE 9:** Hydrogen binding during the simulation of the RELA-luteolin system.

Acceptor^a^	DonorH^b^	Donor^c^	Frac^d^	AvgDist^e^	AvgAng^f^
Ligand@O3	HIE_40@HE2	HIE_40@NE2	0.3152	3.0903	148.5026
GLN_96@O	Ligand@H9	Ligand@O2	0.2636	2.6842	162.5045
Ligand@O5	LYS_38@H	LYS_38@N	0.227	3.1853	158.6617
ASN_97@OD1	Ligand@H10	Ligand@O4	0.1923	2.7355	164.0721

^a^ Acceptor, hydrogen bond acceptors.

^b^ Acceptor hydrogen donor.

^c^ Donor, hydrogen bond donor.

^d^ Frac, frequency of occurrence in the simulation.

^e^ AvgDist, average distance of hydrogen bonds.

^f^ AvgAng, average bond angle of hydrogen bonds.

**TABLE 10:** Hydrogen binding during the simulation of the RELA-quercetin system.

Acceptor	DonorH	Donor	Frac	AvgDist	AvgAng
Ligand@O2	HIE_40@HE2	HIE_40@NE2	0.6069	3.0949	153.2802
SER_368@OXT	Ligand@H9	Ligand@O5	0.4302	2.6539	164.0381
SER_27@OG	Ligand@H8	Ligand@O2	0.4083	2.868	147.5835
SER_368@O	Ligand@H9	Ligand@O5	0.3361	2.6502	163.8601
Ligand @O7	LYS_38@H	LYS_38@N	0.2677	3.2296	155.2125

^a^ Acceptor, hydrogen bond acceptors.

^b^ Acceptor hydrogen donor.

^c^ Donor, hydrogen bond donor.

^d^ Frac, frequency of occurrence in the simulation.

^e^ AvgDist, average distance of hydrogen bonds.

^f^ AvgAng, average bond angle of hydrogen bonds.

Overall, both RELA-luteolin and RELA-quercetin show good kinetic properties, indicating the correct docking pose. Both luteolin and quercetin fall in small conformational space domains with stable kinetic characteristics of the systems. In addition, they both have strong binding energies and stable hydrogen bond interactions. The results showed that both components luteolin and quercetin of P. vulgaris could stably bind NF-κB, which suggested that P. vulgaris could ameliorate COVID-19 AKI through NF-κB.

## Discussion

The incidence of COVID-19-associated acute kidney injury (AKI) has received increasing attention, particularly in severe cases. Traditional Chinese Medicine (TCM) has made significant contributions to the medical field, with reported preventative and therapeutic effects against COVID-19 and AKI (Li et al., 2019; Huang Y. F. et al., 2020; Gajewski et al., 2021). In this study, we developed a *P. vulgaris* target COVID-19–related gene set comprising of 31 genes. Analysis using GO and KEGG revealed that *P. vulgaris* can regulate the inflammatory response process and virus defence mechanism. Furthermore, we identified eight key target genes from the total of 31 genes via PPI network and GO and KEGG network analyses. Amongst all the molecules, RELA-encoded protein (NF-κB) showed the highest docking scores; therefore, it was chosen for MD simulations. These results can potentially promote basic research on SARS-CoV-2 infection and help with target drug design.

*P. vulgaris* is a traditional Chinese herb used in the treatment of various kidney diseases, including diabetic nephropathy and AKI (Bai et al., 2016; Peng et al., 2016; Namgung et al., 2017; Wu et al., 2020). Studies have shown that *P. vulgaris* can inhibit SARS-CoV-2, preventing it from entering cells by blocking the binding of the virus to the ACE2 receptor using its aqueous extract (APV) (Ao et al., 2021). Therefore, we hypothesize that *P. vulgaris* may be pharmacologically effective in treating COVID-19 AKI due to its inhibitory effects on SARS-CoV-2 and protective effects on the kidney. According to the principles of network pharmacology, we first identified effective active compounds of *P. vulgaris* that may ameliorate COVID-19 AKI. Initially, 11 active molecules of *P. vulgaris* were selected from the TCM database based on OB and DL, followed by the construction of an ingredients-targets network map and molecular docking, three ingredients including quercetin, luteolin and kaempferol were found most significant, as they showed revealed strong interactions with the previously mentioned eight key target proteins. The three identified molecules belong to the group of flavonoid steroids (Kang et al., 2011; Yu et al., 2020; Derosa et al., 2021) and are potential compounds for the treatment of COVID-19 (Luo et al., 2020; Yu et al., 2020; Derosa et al., 2021). Quercetin, luteolin and kaempferol play important roles in the activity of several TCMs against COVID-19. A clinical trial found that quercetin is safe and effective in lowering the serum levels of ALP, q-CRP, and LDH as critical markers involved in COVID-19 severity (Shohan et al., 2022). In addition, their renal protective effects have also been verified in several studies, wherein quercetin has been shown to ameliorate renal injury by inhibiting iron death as well as downregulating the phosphorylation of NF-κB (Tan et al., 2020; Wang et al., 2021), luteolin by regulating P53-dependent tubular apoptosis (Kang et al., 2011), and kaempferol by improving cell injury, oxidative stress and inflammation (Yuan et al., 2021).

Combined with the PPI network and the GO and KEGG-target network, eight key target genes of *P. vulgaris* against COVID-19 AKI were identified, including IL6, VEGFA, CXCL8, IL1B, ICAM1, CCL2, IL10 and RELA. The expression level of these eight genes was strongly associated with COVID-19 AKI. The secretion of cytokines and chemokines, including IL6, CXCL8, IL1B, IL10 and CCL2, is significantly increased in COVID-19 AKI. These inflammatory factors participate in the cytokine storm and act as the important agents causing renal injury (Ahmadian et al., 2021). Studies have shown that IL6 is the most critical mediator in COVID-19 and is closely associated with poor prognosis and mortality (Coomes and Haghbayan, 2020). Excessive IL6 signaling causes several biological effects, such as induction of vascular endothelial growth factor (VEGF) expression, thereby increasing vascular permeability, hyperactivation of T-helper 17 (Th17) cells, and induction of effector T-cell death, resulting in kidney injury (Nechemia-Arbely et al., 2008; Liu et al., 2020). The expression levels of CXCL8, IL1B, CCL-2 and IL10 are also significantly upregulated after SARS-CoV-2 infection and are related to the severity of the disease. CXCL8 control the chemotactic activity of neutrophils and monocytes, IL1B increase the production of IL6 and participate in the differentiation of Th17 cells, CCL-2/MCP-1 can chemoattract monocytes, and the expression of IL10, an anti-inflammatory factor, are feedback increased. These cytokines can result in AKI by causing excessive inflammatory responses (Coperchini et al., 2020; Ahmadian et al., 2021). ICAM1 promotes leukocyte migration through the endothelium, which leads to the infiltration of inflammatory cells and aggravates tissue damage (Ahmadian et al., 2021). VEGFA, a marker of vascular endothelial cell damage, is regulated by hypoxia-inducible factor (HIF). VEGFA is primarily involved in angiogenesis and vascular permeability and is significantly increased in patients with COVID-19, wherein it correlates with the disease severity (Rovas et al., 2021). In addition, TGFB1 is also a related target in the 31genes obtained by intersecting drug target genes and disease-related genes, which may regulate the smad3 activation. As SARS-CoV-2 N protein can enhance TGF-β/Smad3 signaling to cause tubular epithelial cell death and AKI via the G1 cell cycle arrest mechanism, the regulation of TGFB1 might be significant in COVID-19 AKI (Wang et al., 2022).

Molecular docking results revealed that quercetin could bind to all eight key target proteins; luteolin could bind to RELA, ICAM1, VEGFA, IL6 and CXCL8, and kaempferol could bind to ICAM1. Quercetin, luteolin and kaempferol have been reported to have anti-inflammatory and anti-oxidative properties. They can downregulate the production of ICAM1 and MCP-1 to reduce the infiltration of mononuclear cells and inhibit the expression of inflammatory cytokines IL6, TNF-α, IL8 and IL1B through the NF-κB signaling pathway (Li et al., 2016; Hu et al., 2019). In addition, these compounds can reduce ROS production and VEGF protein concentrations through the Nrf2/HO-1 signaling pathway to alleviate oxidative stress and improve vascular function (Jia et al., 2015; Boeing et al., 2020; Yao et al., 2020; Ozyel et al., 2021). Therefore, it is reasonable to say that *P. vulgaris* can modulate the cytokine storm and improve inflammation, oxidative stress and vascular endothelial function in patients with COVID-19 by targeting the eight core proteins, thereby reducing renal injury.

GO and KEGG pathway analysis revealed that drug-disease intersection genes were substantially enriched in virus-related functions and pathways, which verified the ability of *P. vulgaris* against viruses. GO enrichment mainly focused on inflammation, cytokines, oxidative stress and angiogenesis, among other factors, which coincided with the biological effects of the eight key target genes described earlier. Analysis of the top 20 KEGG signaling pathways revealed that these target genes were mainly enriched in HIF-1, VEGF, NF-κB, JAK/STAT, Th17 cell differentiation, TNF-α and Nrf2 signaling pathways. NF-κB signaling pathway was enriched to the most number of key target genes, and based on its significant effect on COVID-19 (Hariharan et al., 2021), it can be inferred that it is the most important signaling pathway of *P. vulgaris* in antagonising COVID-19 AKI. The effects are accomplished by anti-oxidative and anti-inflammatory actions, improvement in the function of blood vessels, and by reducing inflammatory factors. Th17 cells also play an important role in the pathogenesis of COVID-19. These cells can promote neutrophil migration and reduce Treg responses by releasing cytokines, such as IL-17 and GM-CSF and thus exacerbate inflammation. This is also a key mechanism causing kidney injury (Martonik et al., 2021).

Because of the highest docking score of RELA-encoded protein (NF-κB) with luteolin and quercetin, NF-κB was selected to perform the MD simulation to further examine the binding effect of NF-κB with luteolin and quercetin, respectively, which verified the result of molecular docking. And the MD simulation results suggested good binding stability. In most cells, NF-κB complexes remain inactive and are mainly located in the cytoplasm where they interact with inhibitory IκB proteins. However, when signaling pathways become activated, the IκB protein is degraded, allowing NF-κB dimers to translocate into the nucleus and regulate the expression of target genes (Hayden and Ghosh, 2011). RELA encodes NF-κB, which can be activated by SARS-CoV-2 Viral DNA and RNA, ROS and cytokines, such as TNF-α, IL1B and IL6, can cause nuclear translocation of NF-κB. (Hariharan et al., 2021). NF-κB can participate in host immunity, inflammation, and regulation of proliferation, differentiation, and apoptosis of B cell and T lymphocytes (Hayden and Ghosh, 2011). Activated NF-κB promotes the expression of multiple cytokines (IL1, IL2, IL6, IL12, TNF-α, LT-α, LT-1β and GM-CSF), chemokines (IL8, MIP-1 and MCP-1), adhesion molecules (ICAM, VCAM and E-selectin), and induces effector enzymes (inducible nitric oxide synthase [iNOS]) (Hariharan et al., 2021), which can aggravate the cytokine storm and oxidative stress on the kidney. As mentioned in the manuscript, NF-κB pathway is a major pathway in this study, which can regulate the expression of other core protein, such as IL6. And the inhibition of NF-κB pathway has the potential to reduce the effect of cytokine storm and improve oxidative stress and vascular function. In summary, NF-κB may be the most potential target for the therapy of *P. vulgaris* on COVID-19 AKI.

In the present study, we investigated the potential therapeutic mechanism of the Chinese medicine *P. vulgaris* in COVID-19 AKI based on network pharmacology and molecular docking approaches. Our findings indicated that NF-κB might be the potent pharmacological target of *P. vulgaris* against COVID-19 induced AKI. In addition, we provided several potential targets for COVID-19 AKI treatment, mainly IL6, VEGFA and RELA, which may help to develop new therapeutic strategies. However, as an *in silico* study, our results have not been experimentally verified. But it is still valuable which can and can provide guidance for drug design and further investigations warranted by the study, such as biological studies on binding affinity and activities of target proteins; preclinical and clinical efficacy and toxicity, *etc.*

## Data Availability

The original contributions presented in the study are included in the article/Supplementary Materials, further inquiries can be directed to the corresponding authors.
