# Supplementary figures and images for: In silico evidence implicating novel mechanisms of Prunella vulgaris L. as a potential botanical drug against COVID-19-associated acute kidney injury
Source: Front Pharmacol. 2023 May 18;14:1188086. doi: 10.3389/fphar.2023.1188086 (PMC10232756; doi:10.3389/fphar.2023.1188086)

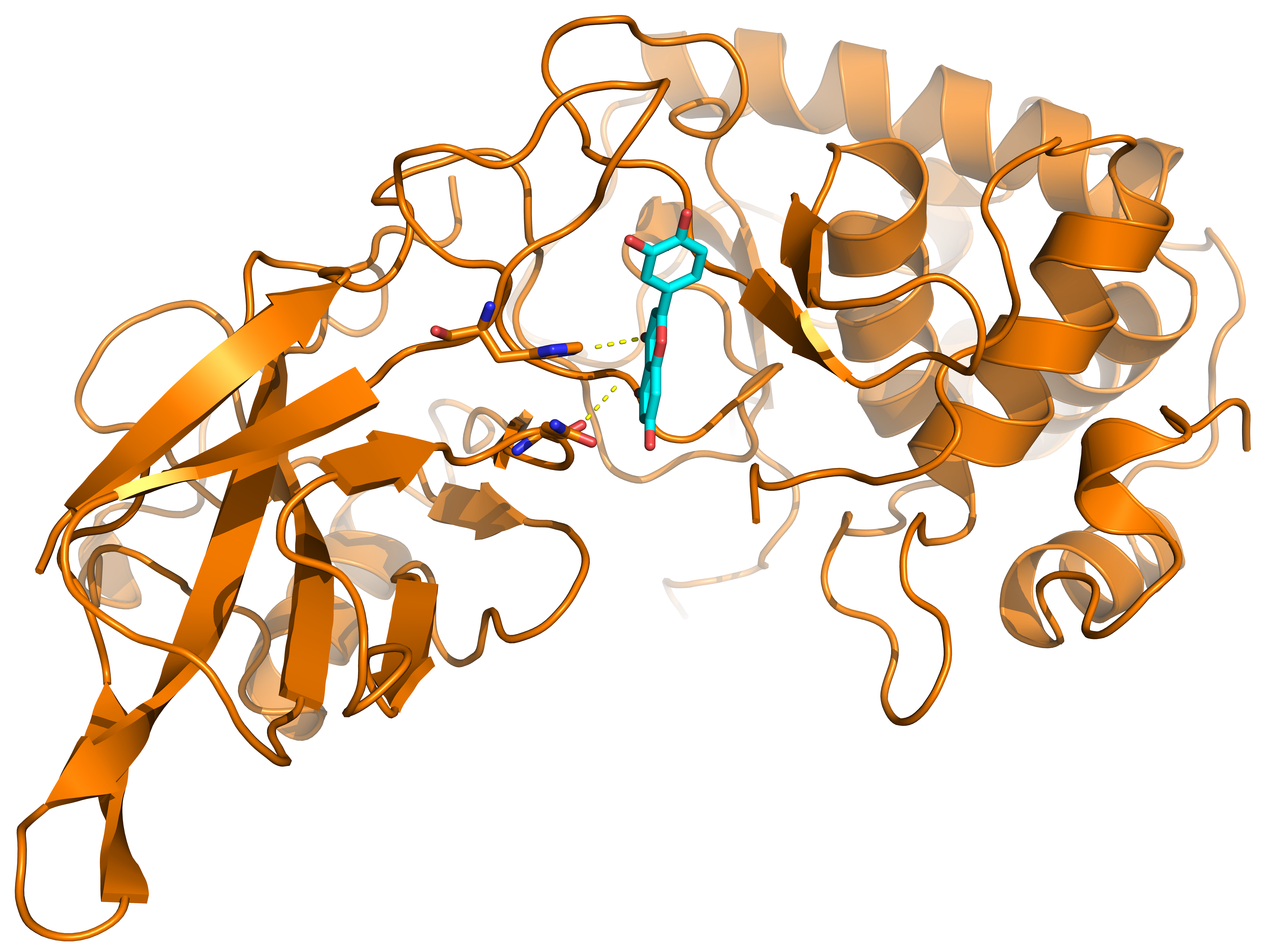

Supplement: Supplementary file 2 [file DataSheet4.ZIP › Molecular dynamics simulation data/RELA_Luteolin_complex/hb-L.png]

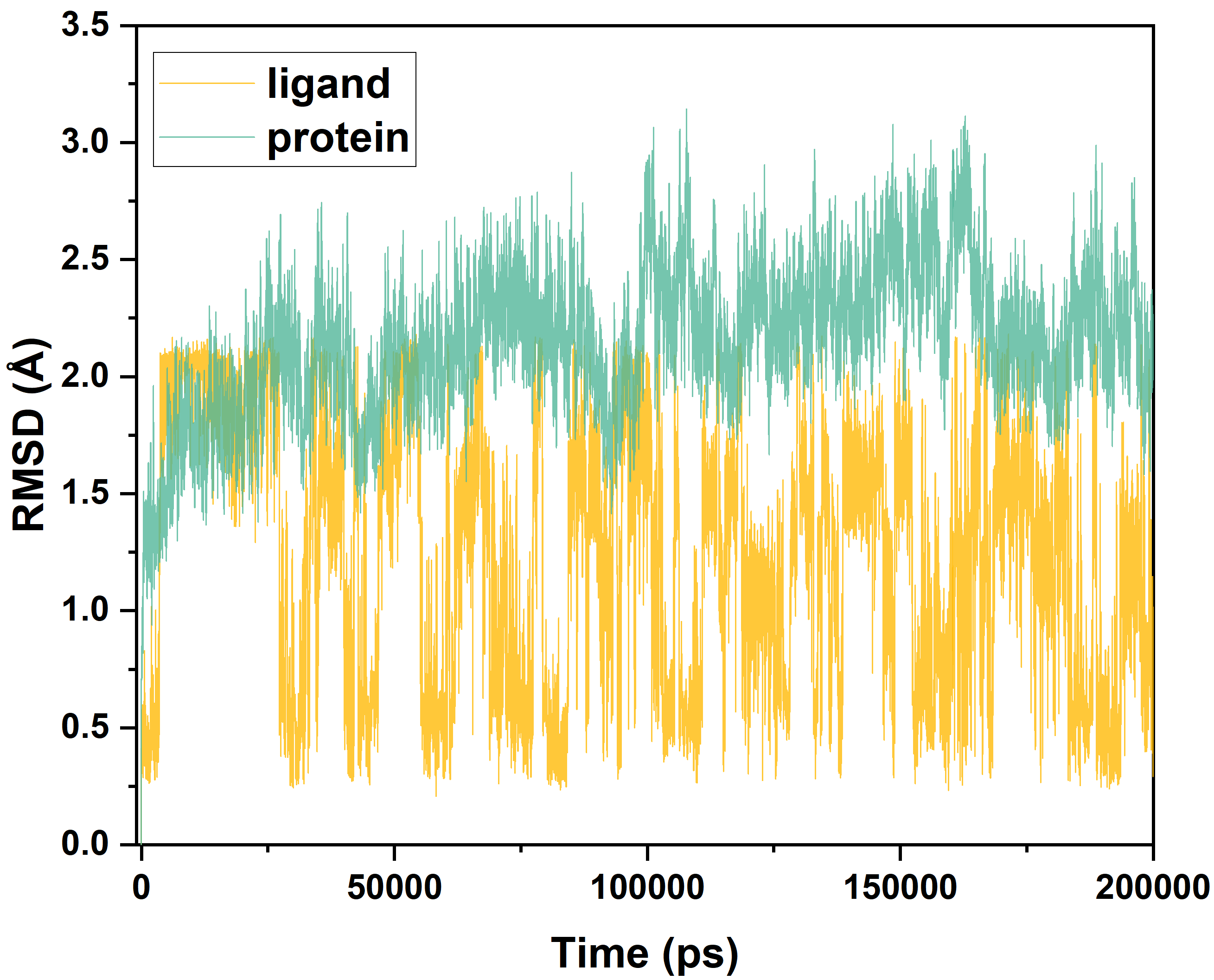

Supplement: Supplementary file 2 [file DataSheet4.ZIP › Molecular dynamics simulation data/RELA_Luteolin_complex/RMSD.tif]

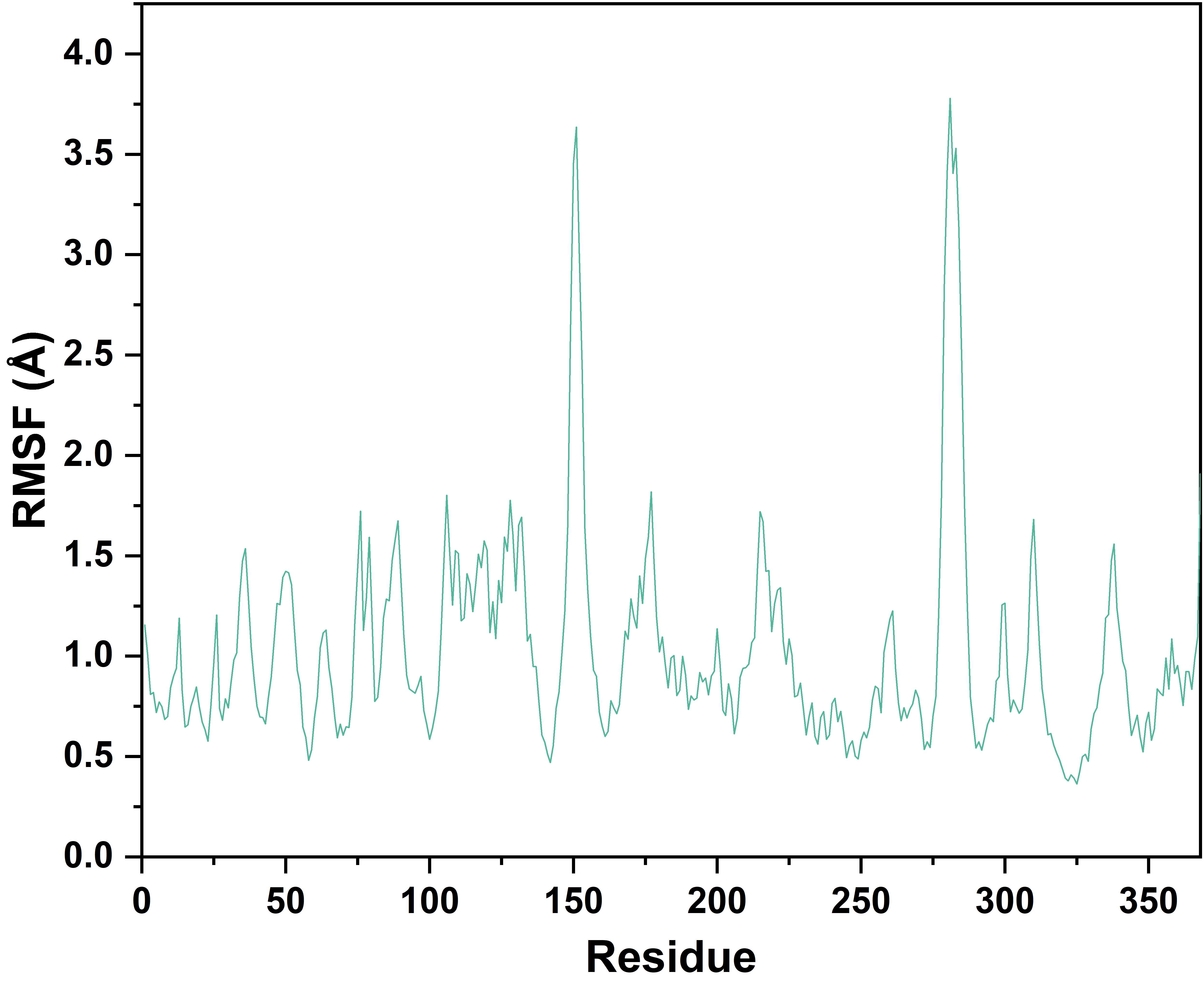

Supplement: Supplementary file 2 [file DataSheet4.ZIP › Molecular dynamics simulation data/RELA_Luteolin_complex/RMSF-L.tif]

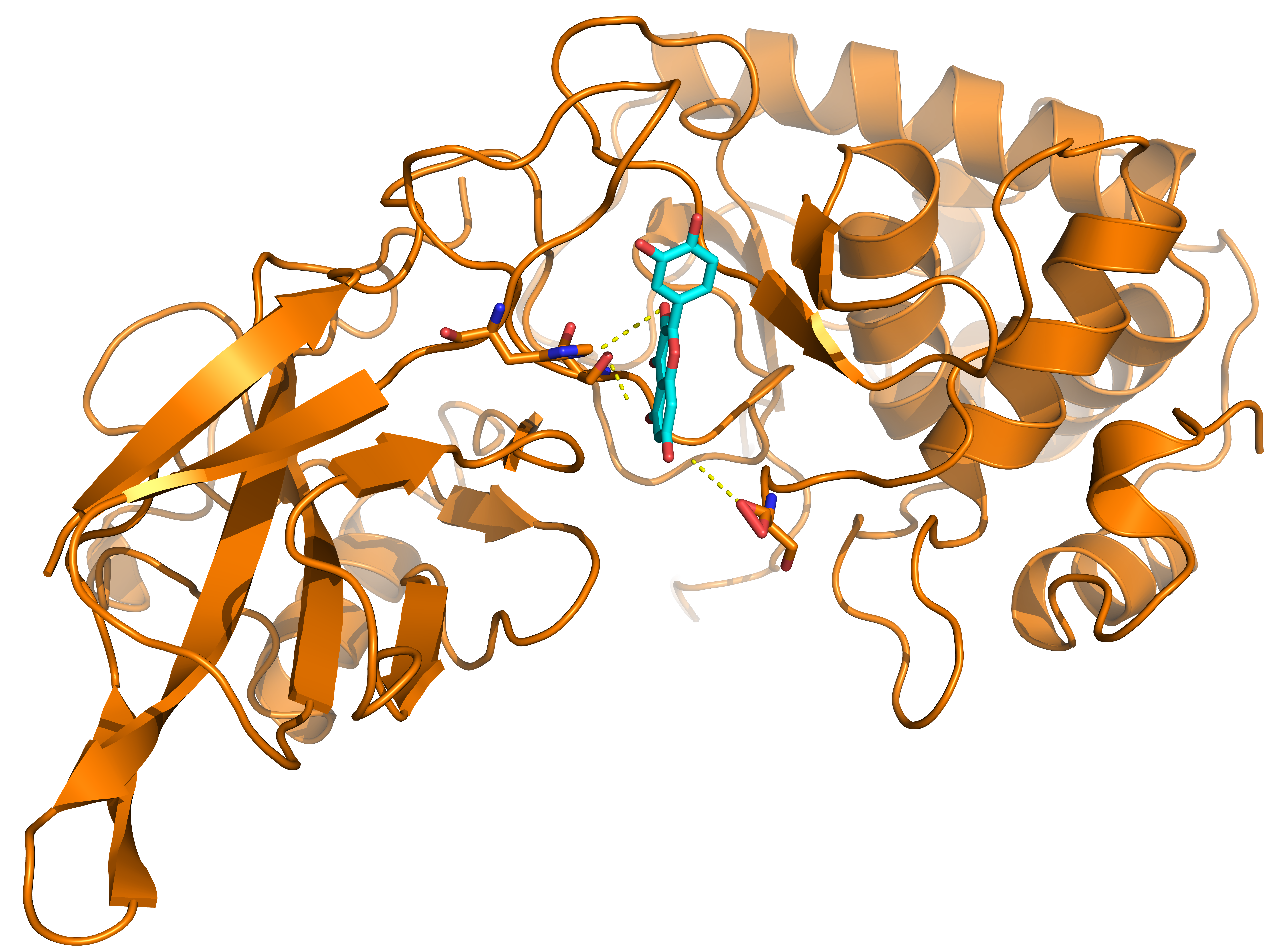

Supplement: Supplementary file 2 [file DataSheet4.ZIP › Molecular dynamics simulation data/RELA_Quercetin_complex/hb-Q.png]

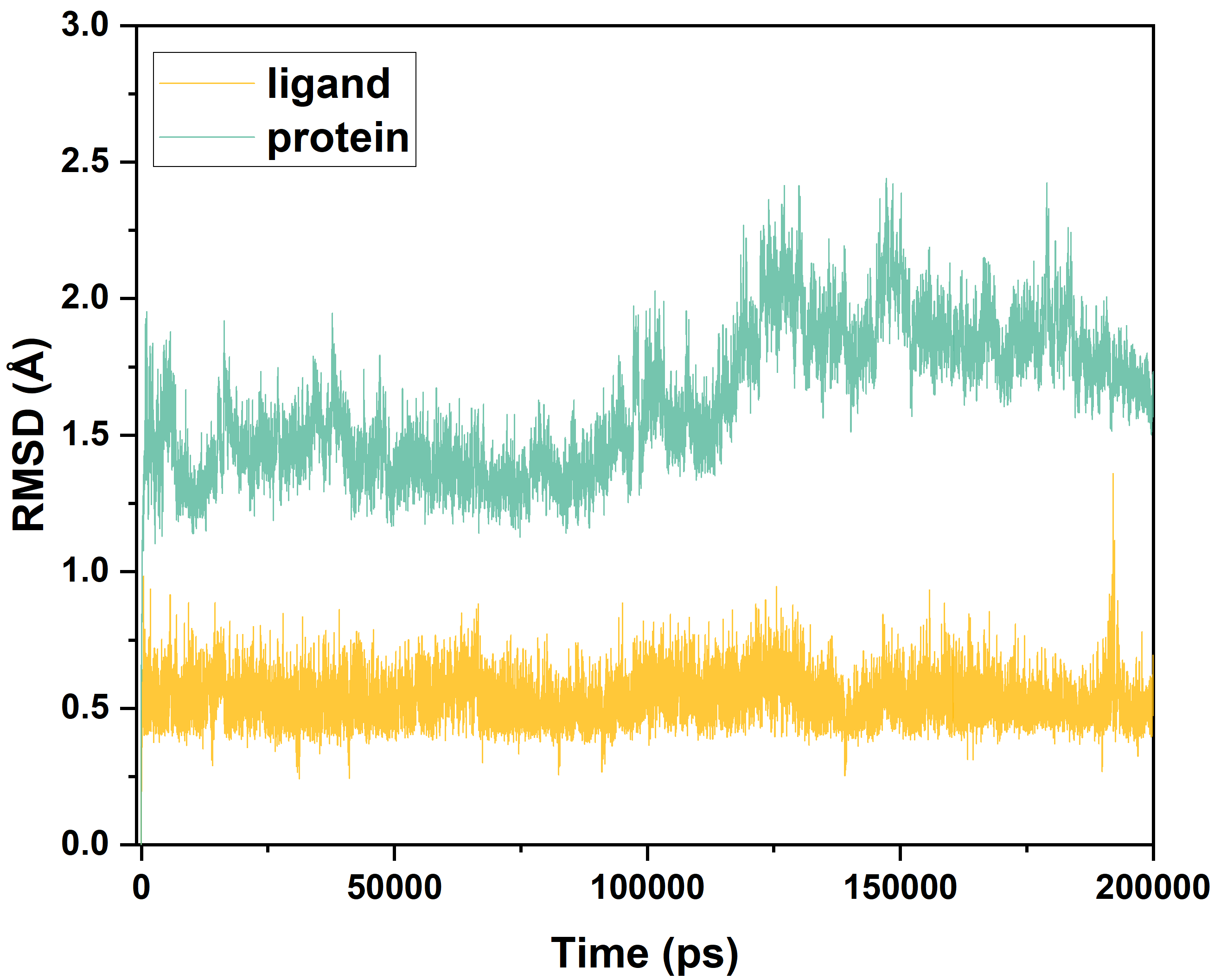

Supplement: Supplementary file 2 [file DataSheet4.ZIP › Molecular dynamics simulation data/RELA_Quercetin_complex/RMSD.tif]

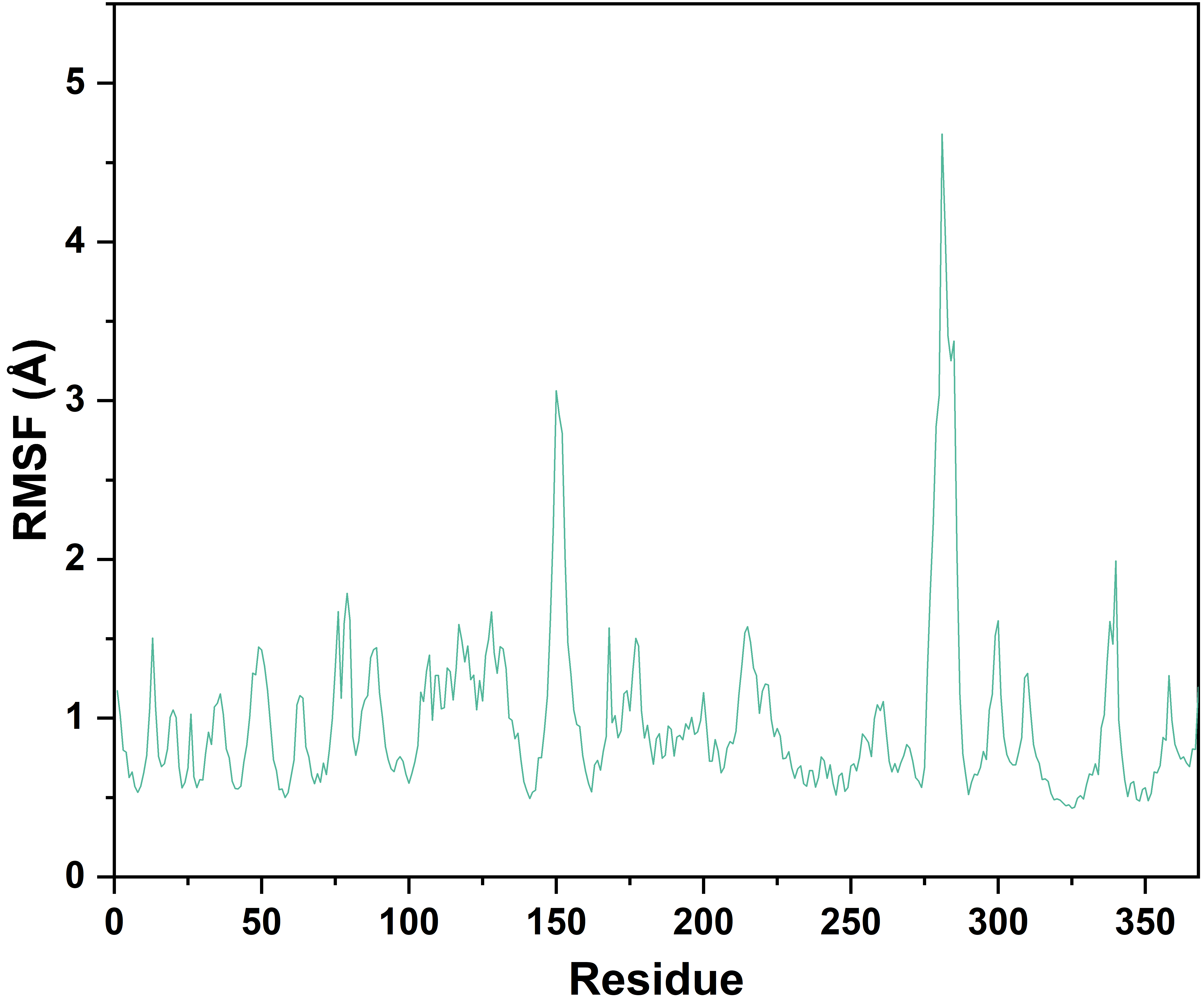

Supplement: Supplementary file 2 [file DataSheet4.ZIP › Molecular dynamics simulation data/RELA_Quercetin_complex/RMSF-Q.tif]

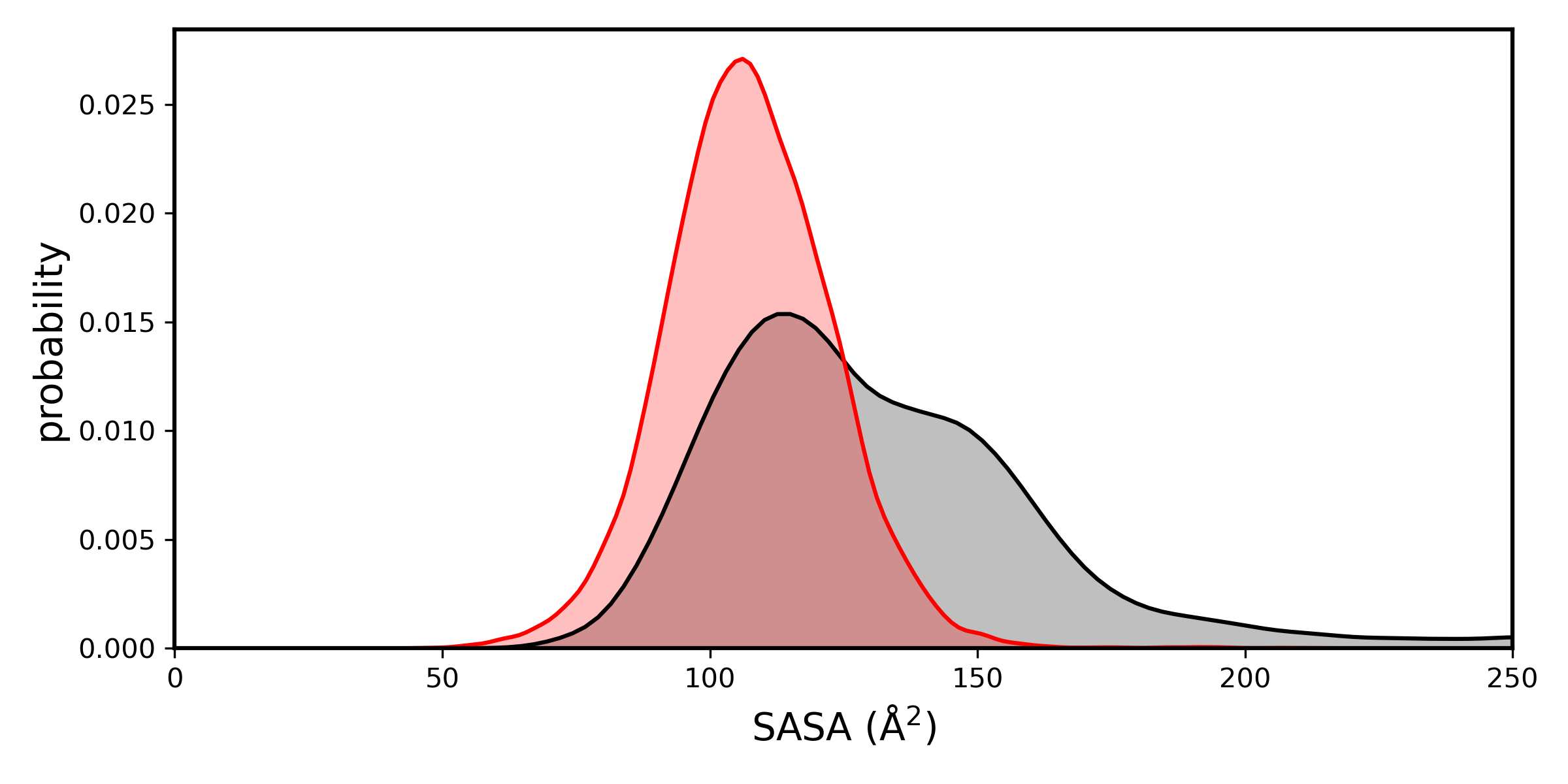

Supplement: Supplementary file 2 [file DataSheet4.ZIP › Molecular dynamics simulation data/SASA.tif]
