# Supplementary material for: In silico evidence implicating novel mechanisms of Prunella vulgaris L. as a potential botanical drug against COVID-19-associated acute kidney injury
Source: Front Pharmacol. 2023 May 18;14:1188086. doi: 10.3389/fphar.2023.1188086 (PMC10232756; doi:10.3389/fphar.2023.1188086)
Supplement: Supplementary file 3 [file DataSheet6.ZIP › R-DATA/R.CODE-GO.KEGG.docx]

rm(list = ls())

rt<-read.csv("crossed_31genes.csv",sep="\t",header=T,check.names=F)

head(rt)

library(ggplot2)

library(clusterProfiler)

library(AnnotationDbi)

library(org.Hs.eg.db)

library("clusterProfiler")

library("enrichplot")

library("pathview")

library("ggnewscale")

library("DOSE")

library(stringr)

library("ggnewscale")

genes=as.vector(rt[,1])

entrezIDs <- mget(genes, org.Hs.egSYMBOL2EG, ifnotfound=NA)

entrezIDs <- as.character(entrezIDs)

rt=cbind(rt,entrezID=entrezIDs)

colnames(rt)=c("symbol","entrezID")

rt=rt[is.na(rt[,"entrezID"])==F,]

gene=rt$entrezID

gene=unique(gene)

id_list<-gene

go <- enrichGO(gene = id_list,

OrgDb = org.Hs.eg.db,

keyType = "ENTREZID",

ont = "ALL",

pAdjustMethod = "BH",

pvalueCutoff = 0.05,

readable = T

go.res <- data.frame(go)

write.csv(go.res,"Table_GO_result.csv")

go.res$enrichment_fold=apply(go.res,1,function(x){

GeneRatio=eval(parse(text=x["GeneRatio"]))

BgRatio=eval(parse(text=x["BgRatio"]))

enrichment_fold=round(GeneRatio/BgRatio,2)

enrichment_fold})

View(go.res)

goBP <- subset(go.res,subset = (ONTOLOGY == "BP"))[1:15,]

goCC <- subset(go.res,subset = (ONTOLOGY == "CC"))[1:15,]

goMF <- subset(go.res,subset = (ONTOLOGY == "MF"))[1:15,]

#if(nrow(GO)<30){

showNum=nrow(GO)}

go.df <- rbind(goBP,goCC,goMF)

dim(goCC)

View(goCC)

write.csv(go.df,"GO_top15.csv",quote = F)

write.csv(goBP,"BP_top15.csv",quote = F)

write.csv(goCC,"CC_top15.csv",quote = F)

write.csv(goMF,"MF_top15.csv",quote = F)

save(rt,goBP,goCC,goMF,go.df,go.res,file = 'GO-31genes')

load(file = 'GO-31genes')

load(file = 'GO-31genes')

go.df<-read.csv("GO_top15.csv",header=T,check.names=F)

go.df$Description <- factor(go.df$Description,levels = rev(go.df$Description))

go.df<-go.df[,(-1)]

head(go.df)

View(go.df)

dim(go.df)

go_bar <- ggplot(data = go.df,

aes(x =Description , y = Count,fill = ONTOLOGY))+

geom_bar(stat = "identity",width = 0.5)+

coord_flip()+theme_bw()+

scale_x_discrete(labels = function(x) str_wrap(x,width = 50))+

labs(x = "GO terms",y = "GeneNumber",title = "Barplot of Enriched GO Terms")）

ggsave(go_bar,filename = "GO_Barplot.pdf",width = 9,height = 7)

barplot(go.df,drop=T)

dotplot(go,showCategory=20)

# KEGG

library(R.utils)

R.utils::setOption( "clusterProfiler.download.method",'auto')

#R.utils::setOption( "clusterProfiler.download.method",'wininet')

#"wininet","auto","libcurl","wget","curl"

kegg <- enrichKEGG(gene = id_list,

organism = "hsa",keyType = "kegg",

pAdjustMethod = "BH",pvalueCutoff = 0.05,qvalueCutoff = 0.05,

minGSSize = 10,maxGSSize = 500,use_internal_data = F)

kegg <- setReadable(kegg,OrgDb = "org.Hs.eg.db",keyType = "ENTREZID")

kegg.df <- data.frame(kegg) # 或kegg.df <- data.frame(kk) # 结果转化成数据框

kegg.df$enrichment_fold=apply(kegg.df,1,function(x){

GeneRatio=eval(parse(text=x["GeneRatio"]))

BgRatio=eval(parse(text=x["BgRatio"]))

enrichment_fold=round(GeneRatio/BgRatio,2)

enrichment_fold})

keggtop20 <- subset(kegg.df[1:20,])

write.csv(kegg.df,"KEGG_Table.csv")

write.csv(keggtop20,"KEGG_top20.csv")

save(rt,id_list,kegg.df,keggtop20,file = 'KEGG-31genes')

keggId="hsa04659"

load(file = 'kegg-31genes')

#plot

keggtop20$number <- factor(keggtop20$Description,levels = rev(keggtop20$Description))

keggtop20$enrichment_fold=apply(keggtop20,1,function(x){

GeneRatio=eval(parse(text=x["GeneRatio"]))

BgRatio=eval(parse(text=x["BgRatio"]))

enrichment_fold=round(GeneRatio/BgRatio,2)

enrichment_fold})

pkegg <-ggplot(data = keggtop20,

aes(x =enrichment_fold,y = reorder(number,Count)))+ #横纵坐标及排序

geom_point(aes(size = Count,color =-1*log10(qvalue)))+ # 气泡大小及颜色设置

theme_bw()+ # 去除背景色

scale_colour_gradient(low = "blue",high = "red")+ # 设置气泡渐变色

labs(size="geneCounts",x = "enrichment fold", y = "pathway name",

title = "Dotplot of Enriched KEGG Pathways"))

ggsave(pkegg,filename = "KEGG_dotplo.pdf")
