# Supplementary material for: In silico evidence implicating novel mechanisms of Prunella vulgaris L. as a potential botanical drug against COVID-19-associated acute kidney injury
Source: Front Pharmacol. 2023 May 18;14:1188086. doi: 10.3389/fphar.2023.1188086 (PMC10232756; doi:10.3389/fphar.2023.1188086)
Supplement: Supplementary file 4 [file DataSheet1.PDF]

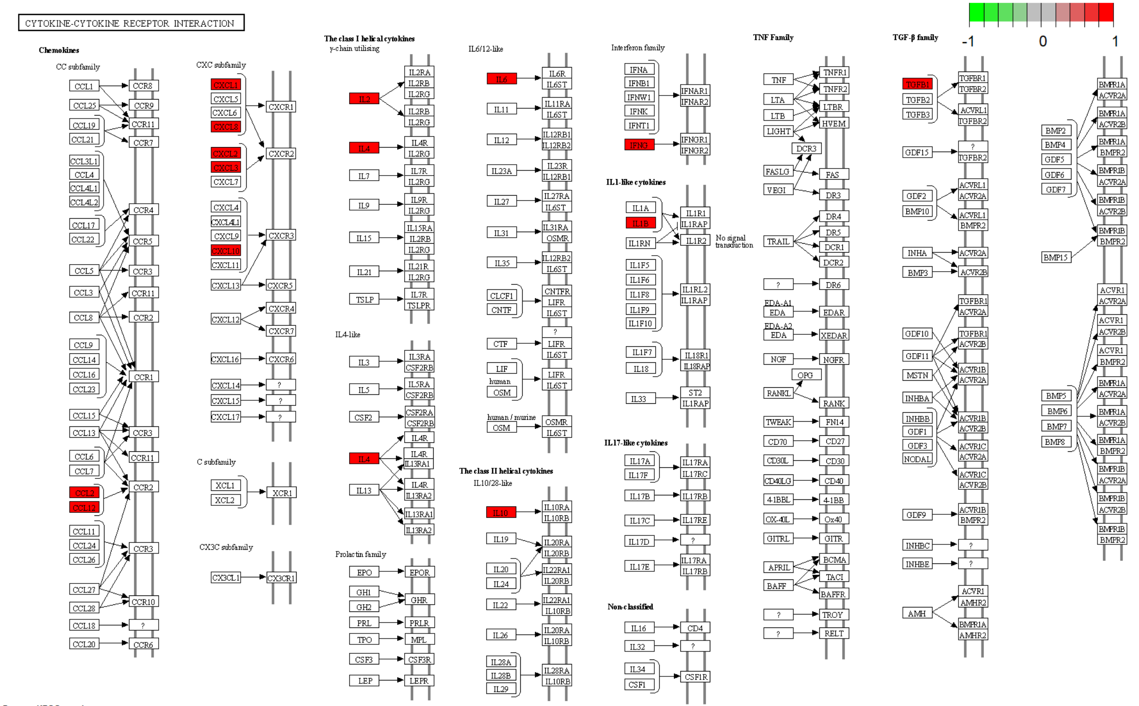

Data on KEGG graph
Rendered by Pathview

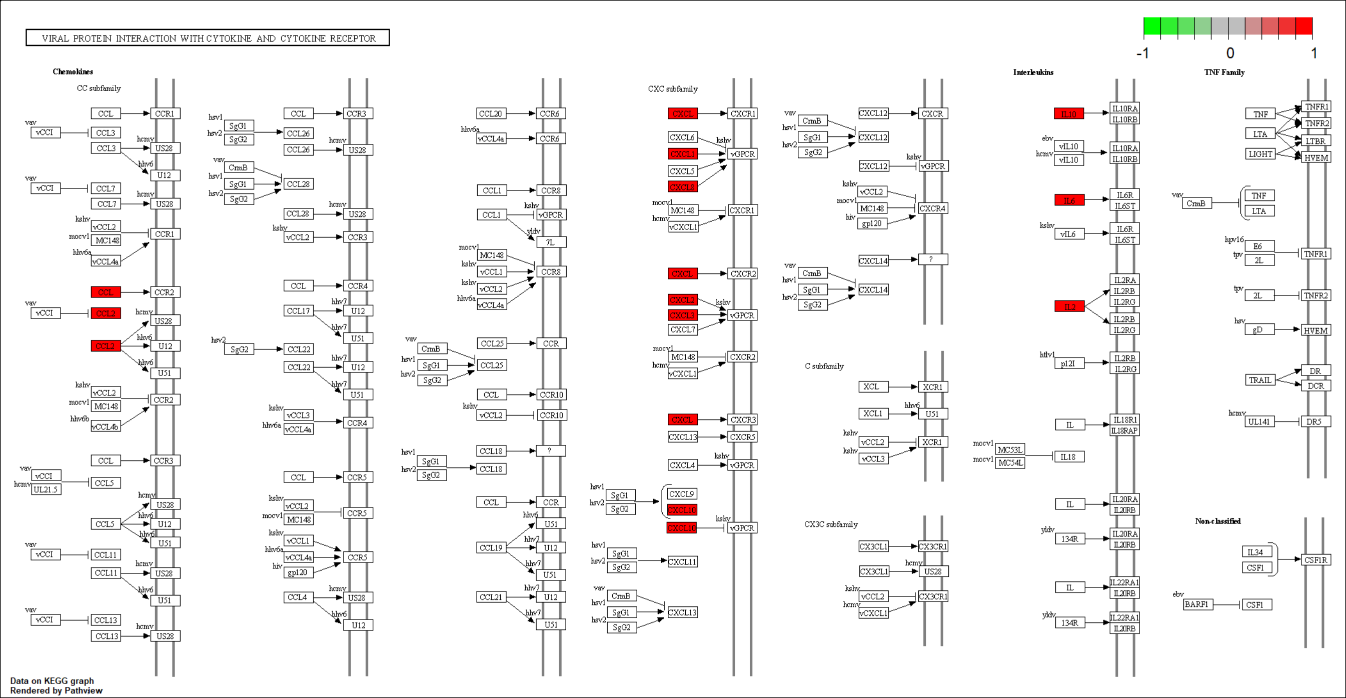

VIRAL PROTEIN INTERACTION WITH CYTOKINE AND CYTOKINE RECEPTOR
-1
0
1
Chemokines
CC subfamily
CCL CCR1
vav vCCI CCL3
CCL3 hcmv US28
hhv6 U12
vav vCCI CCL7
CCL7 hcmv US28
kshv vCCL2
mocv1 MC148 CCR1
hhv6a vCCL4a
CCL CCR2
vav vCCI CCL2
hcmv US28
CCL2 hhv6 U12
hhv6 U51
kshv vCCL2
mocv1 MC148 CCR2
hhv6b vCCL4b
CCL CCR3
vav vCCI
hcmv UL21.5 CCL5
hcmv US28
CCL5 hhv6 U12
hhv6 U51
vav vCCI CCL11
CCL11 hcmv US28
hhv6 U51
vav vCCI CCL13
CCL13 hcmv US28
CCL CCR3
hsv1 SgG1
hsv2 SgG2 CCL26
CCL26 hcmv US28
vav CrmB
hsv1 SgG1 CCL28
hsv2 SgG2
CCL28 hcmv US28
kshv vCCL2 CCR3
CCL CCR4
CCL17 hhv7 U12
hhv7 U51
hsv2 SgG2 CCL22
CCL22 hhv7 U12
hhv7 U51
kshv vCCL3
hhv6a vCCL4a CCR4
CCL CCR5
kshv vCCL2
mocv1 MC148 CCR5
kshv vCCL1
hhv6a vCCL4a CCR5
hiv gp120
CCL4 hcmv US28
hhv6 U12
CCL20 CCR6
hhv6a vCCL4a CCR6
CCL1 CCR8
CCL1 kshv vGPCR
yldv 7L
mocv1 MC148
kshv vCCL1 CCR8
kshv vCCL2
hhv6a vCCL4a
vav CrmB
hsv1 SgG1 CCL25
hsv2 SgG2
CCL25 CCR
CCL CCR10
kshv vCCL2 CCR10
CCL18 ?
hsv1 SgG1
hsv2 SgG2 CCL18
CCL CCR
CCL19 hhv6 U51
hhv7 U12
hhv7 U51
CCL21 hhv7 U12
hhv7 U51
CXC subfamily
CXCL CXCR1
CXCL6 kshv vGPCR
CXCL1
CXCL5
CXCL8
mocv1 MC148 CXCR1
hcmv vCXCL1
CXCL CXCR2
CXCL2 kshv vGPCR
CXCL3
CXCL7
mocv1 MC148 CXCR2
hcmv vCXCL1
CXCL CXCR3
CXCL13 CXCR5
CXCL4 kshv vGPCR
hsv1 SgG1 CXCL9
hsv2 SgG2 CXCL10
CXCL10 kshv vGPCR
hsv1 SgG1
hsv2 SgG2 CXCL11
vav CrmB
hsv1 SgG1 CXCL13
hsv2 SgG2
vav CrmB CXCL12 CXCR
hsv1 SgG1 CXCL12
hsv2 SgG2
CXCL12 kshv vGPCR
kshv vCCL2
mocv1 MC148 CXCR4
hiv gp120
CXCL14 ?
vav CrmB
hsv1 SgG1 CXCL14
hsv2 SgG2
C subfamily
XCL XCR1
XCL1 hhv6 U51
kshv vCCL2 XCR1
kshv vCCL3
CX3C subfamily
CX3CL1 CX3CR1
CX3CL1 hcmv US28
kshv vCCL2 CX3CR1
hcmv vCXCL1
Interleukins
IL10 IL10RA IL10RB
ebv vIL10 IL10RA
hcmv vIL10 IL10RB
IL6 IL6R IL6ST
kshv vIL6 IL6R IL6ST
IL2RA IL2RB IL2RG
IL2 IL2RB IL2RG
htlv1 p12I IL2RB IL2RG
IL IL18R1 IL18RAP
mocv1 MC53L
mocv1 MC54L IL18
IL IL20RA IL20RB
yldv 134R IL20RA IL20RB
IL IL22RA1 IL20RB
yldv 134R IL22RA1 IL20RB
TNF Family
TNF TNFR1
LTA TNFR2
LIGHT LTBR
HVEM
vav CrmB TNF
LTA
hpv16 E6
hpv 2L TNFR1
hpv 2L TNFR2
hsv gD HVEM
TRAIL DR
DCR
hcmv UL141 DR5
Non-classified
IL34
CSF1 CSF1R
ebv BARF1 CSF1
Data on KEGG graph
Rendered by Pathview

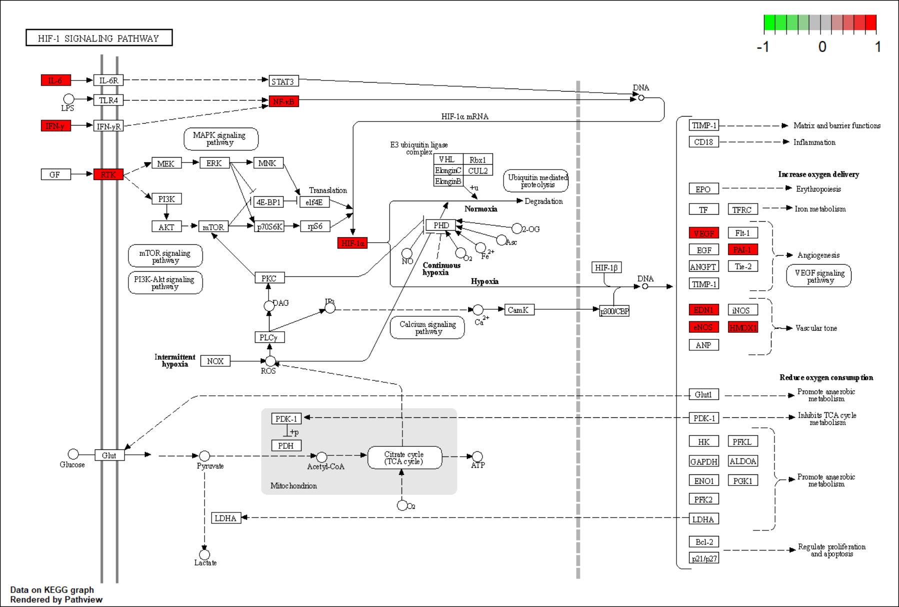
HIF-1 SIGNALING PATHWAY
-1
0
1
IL-6
IL-6R
STAT3
LPS
TLR4
NF-κB
IFN-γ
IFN-γR
DNA
HIF-1α mRNA
MAPK signaling pathway
GF
RTK
MEK
ERK
MNK
PI3K
AKT
mTOR
4E-BP1
eIf4E
p70S6K
rpS6
Translation
E3 ubiquitin ligase complex
VHL
Rbx1
ElonginC
CUL2
ElonginB
+u
Ubiquitin mediated proteolysis
Degradation
Normoxia
PHD
2-OG
Asc
Fe2+
O2
NO
Continuous hypoxia
Hypoxia
HIF-1α
mTOR signaling pathway
PI3K-Akt signaling pathway
PKC
DAG
IP3
PLCγ
Ca2+
CamK
p300/CBP
HIF-1β
Calcium signaling pathway
Intermittent hypoxia
NOX
ROS
TIMP-1
Matrix and barrier functions
CD18
Inflammation
Increase oxygen delivery
EPO
Erythropoiesis
TF
TFRC
Iron metabolism
VEGF
Flt-1
EGF
PAI-1
ANGPT
Tie-2
TIMP-1
Angiogenesis
VEGF signaling pathway
EDN1
iNOS
eNOS
HMOX1
ANP
Vascular tone
Reduce oxygen consumption
Glut1
Promote anaerobic metabolism
PDK-1
Inhibits TCA cycle metabolism
HK
PFKL
GAPDH
ALDOA
ENO1
PGK1
PFK2
LDHA
Promote anaerobic metabolism
Bcl-2
p21/p27
Regulate proliferation and apoptosis
PDK-1
+p
PDH
Glucose
Glut
Pyruvate
Acetyl-CoA
Citrate cycle (TCA cycle)
ATP
Mitochondrion
O2
LDHA
Lactate
Data on KEGG graph
Rendered by Pathview

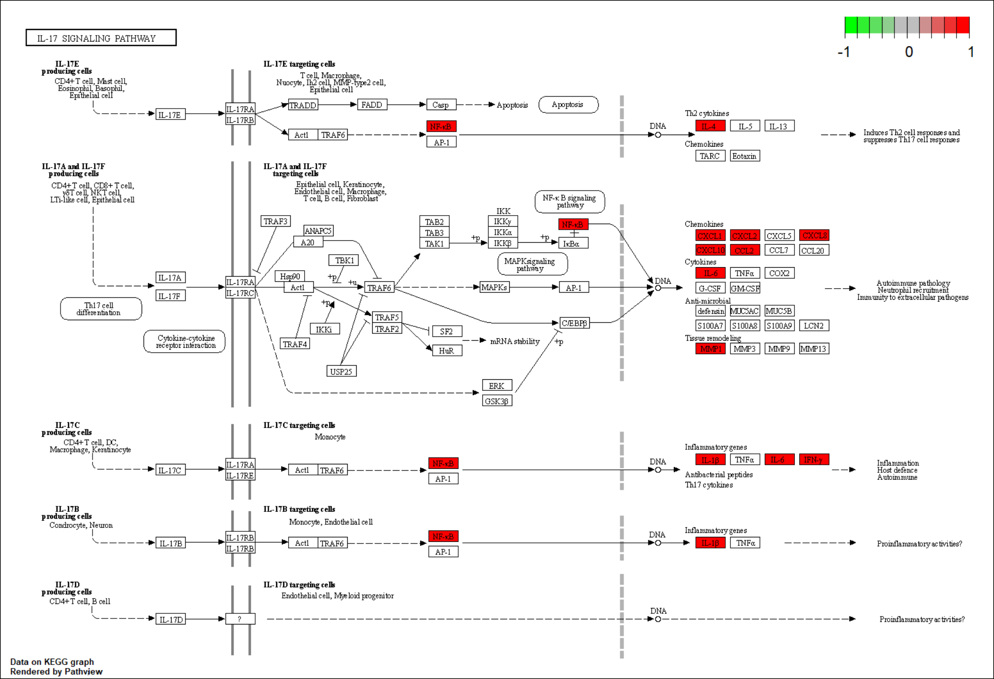

IL-17 SIGNALING PATHWAY
-1
0
1
IL-17E producing cells
CD4+ T cell, Mast cell, Eosinophil, Basophil, Epithelial cell
IL-17E
IL-17RA
IL-17RB
IL-17E targeting cells
T cell, Macrophage, Nuocyte, Ih2 cell, MPP-type2 cell, Epithelial cell
TRADD
FADD
Casp
Apoptosis
Apoptosis
Act1
TRAF6
NF-κB
AP-1
DNA
Th2 cytokines
IL-4
IL-5
IL-13
Chemokines
TARC
Eotaxin
Induces Th2 cell responses and suppresses Th17 cell responses
IL-17A and IL-17F producing cells
CD4+ T cell, CD8+ T cell, γδT cell, NKT cell, LTi-like cell, Epithelial cell
IL-17A
IL-17F
Th17 cell differentiation
Cytokine-cytokine receptor interaction
IL-17RA
IL-17RC
IL-17A and IL-17F targeting cells
Epithelial cell, Keratinocyte, Endothelial cell, Macrophage, T cell, B cell, Fibroblast
TRAF3
ANAPC5
A20
TBK1
Hsp90
Act1
+p
+u
TRAF6
IKKi
TRAF4
USP25
TRAF5
TRAF2
SF2
HuR
mRNA stability
TAB2
TAB3
TAK1
IKK
IKKγ
IKKα
IKKβ
NF-κB signaling pathway
NF-κB
IκBα
MAPKsignaling pathway
MAPKs
AP-1
C/EBPβ
ERK
GSK3β
DNA
Chemokines
CXCL1
CXCL2
CXCL5
CXCL8
CXCL10
CCL2
CCL7
CCL20
Cytokines
IL-6
TNFα
COX2
G-CSF
GM-CSF
Anti-microbial
defensin
MUC5AC
MUC5B
S100A7
S100A8
S100A9
LCN2
Tissue remodeling
MMP1
MMP3
MMP9
MMP13
Autoimmune pathology
Neutrophil recruitment
Immunity to extracellular pathogens
IL-17C producing cells
CD4+ T cell, DC, Macrophage, Keratinocyte
IL-17C
IL-17RA
IL-17RE
IL-17C targeting cells
Monocyte
Act1
TRAF6
NF-κB
AP-1
DNA
Inflammatory genes
IL-1β
TNFα
IL-6
IFN-γ
Antibacterial peptides
Th17 cytokines
Inflammation
Host defence
Autoimmune
IL-17B producing cells
Condrocyte, Neuron
IL-17B
IL-17RB
IL-17RB
IL-17B targeting cells
Monocyte, Endothelial cell
Act1
TRAF6
NF-κB
AP-1
DNA
Inflammatory genes
IL-1β
TNFα
Proinflammatory activities?
IL-17D producing cells
CD4+ T cell, B cell
IL-17D
?
IL-17D targeting cells
Endothelial cell, Myeloid progenitor
DNA
Proinflammatory activities?
Data on KEGG graph
Rendered by Pathview

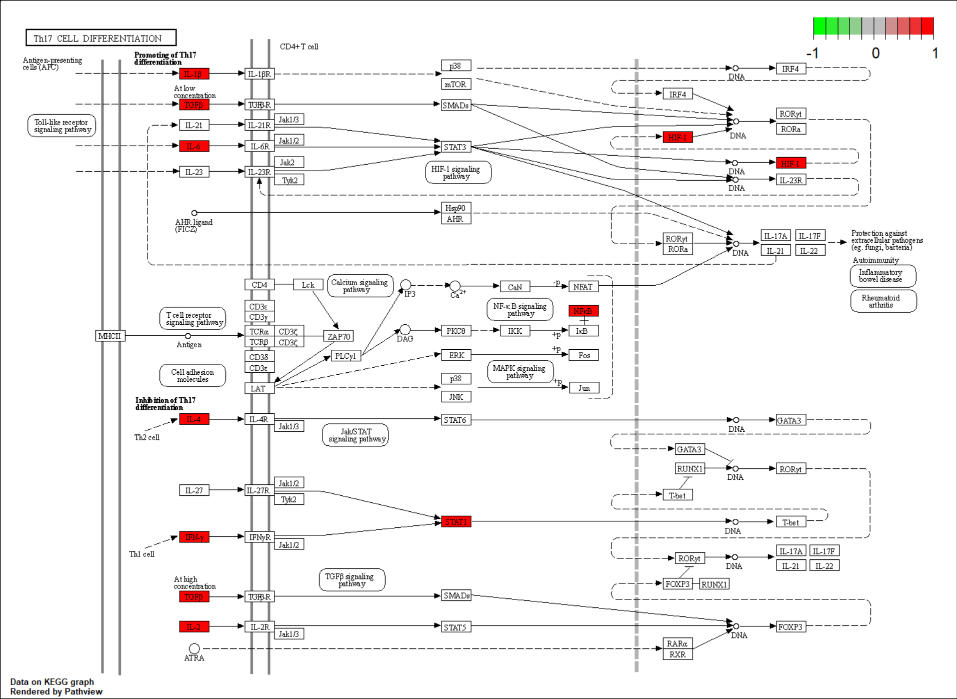

Th17 CELL DIFFERENTIATION
-1
0
1
CD4+ T cell
Antigen-presenting cells (APC)
Promoting of Th17 differentiation
IL-1β
IL-1βR
p38
mTOR
DNA
IRF4
At low concentration
TGFβ
TGFβ-R
SMADs
Toll-like receptor signaling pathway
IL-21
IL-21R
Jak1/3
RORγt
RORα
HIF-1
IL-6
IL-6R
Jak1/2
STAT3
IL-23
IL-23R
Jak2
Tyk2
HIF-1 signaling pathway
IL-23R
AHR ligand (FICZ)
Hsp90
AHR
IL-17A
IL-17F
IL-21
IL-22
Protection against extracellular pathogens (eg. fungi, bacteria)
Autoimmunity
Inflammatory bowel disease
Rheumatoid arthritis
CD4
Lck
Calcium signaling pathway
IP3
Ca2+
CaN
NFAT
CD3ε
CD3γ
T cell receptor signaling pathway
NF-κB signaling pathway
NFκB
MHCII
Antigen
TCRα
TCRβ
CD3ζ
ZAP70
DAG
PKCθ
IKK
IκB
CD3δ
PLCγ1
ERK
Fos
Cell adhesion molecules
LAT
MAPK signaling pathway
Jun
JNK
Inhibition of Th17 differentiation
IL-4
IL-4R
STAT6
GATA3
Th2 cell
Jak/STAT signaling pathway
RUNX1
T-bet
IL-27
IL-27R
Tyk2
STAT1
IFN-γ
IFNγR
Th1 cell
FOXP3
TGFβ signaling pathway
At high concentration
IL-2
IL-2R
STAT5
RARα
RXR
ATRA
Data on KEGG graph
Rendered by Pathview

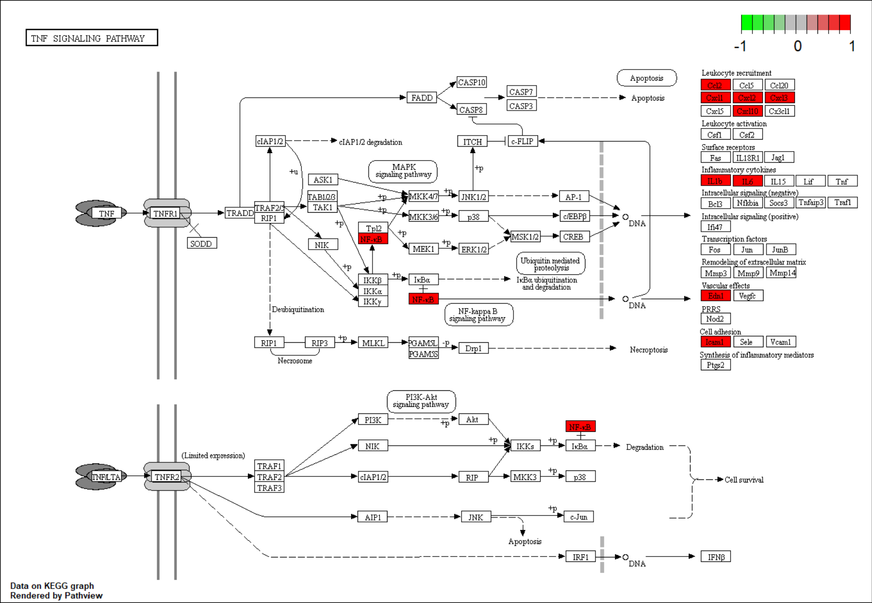
TNF SIGNALING PATHWAY
-1
0
1
TNF
TNFR1
SODD
TRADD
TRAF2/5
RIP1
cIAP1/2
cIAP1/2 degradation
+u
ASK1
TAB1/2/3
TAK1
NIK
MAPK signaling pathway
MKK4/7
MKK3/6
MEK1
JNK1/2
p38
ERK1/2
Tpl2
NF-κB
IKKβ
IKKα
IKKγ
IκBα
NF-κB
FADD
CASP10
CASP8
CASP7
CASP3
Apoptosis
Apoptosis
ITCH
c-FLIP
AP-1
c/EBPβ
MSK1/2
CREB
DNA
DNA
Ubiquitin mediated proteolysis
IκBα ubiquitination and degradation
NF-kappa B signaling pathway
Deubiquitination
RIP1
RIP3
MLKL
PGAM5L
PGAM5S
Drp1
Necroptosis
Necrosome
+p
-p
Leukocyte recruitment
Ccl2
Ccl5
Ccl20
Cxcl1
Cxcl2
Cxcl3
Cxcl5
Cxcl10
Cx3cl1
Leukocyte activation
Csf1
Csf2
Surface receptors
Fas
IL18R1
Jag1
Inflammatory cytokines
IL1b
IL6
IL15
Lif
Tnf
Intracellular signaling (negative)
Bcl3
Nfkbia
Socs3
Tnfaip3
Traf1
Intracellular signaling (positive)
Ifi47
Transcription factors
Fos
Jun
JunB
Remodeling of extracellular matrix
Mmp3
Mmp9
Mmp14
Vascular effects
Edn1
Vegfc
PRRS
Nod2
Cell adhesion
Icam1
Sele
Vcam1
Synthesis of inflammatory mediators
Ptgs2
PI3K-Akt signaling pathway
PI3K
Akt
NIK
IKKs
NF-κB
IκBα
Degradation
Cell survival
(Limited expression)
TNF/LTA
TNFR2
TRAF1
TRAF2
TRAF3
cIAP1/2
RIP
MKK3
p38
AIP1
JNK
c-Jun
Apoptosis
IRF1
DNA
IFNβ
Data on KEGG graph
Rendered by Pathview

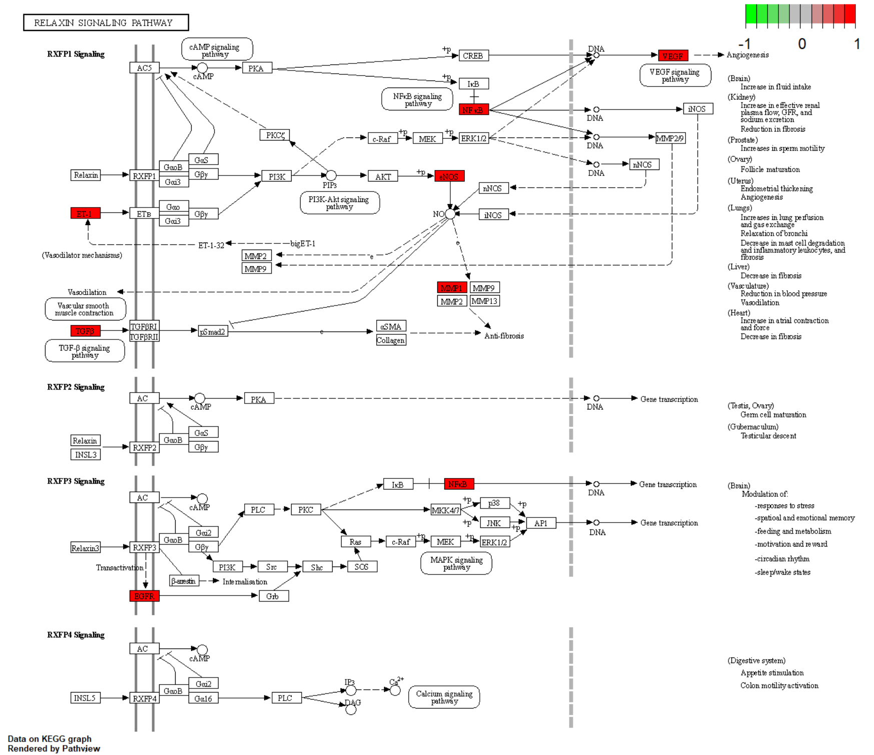
RELAXIN SIGNALING PATHWAY
-1
0
1
RXFP1 Signaling
cAMP signaling pathway
AC5
cAMP
PKA
CREB
IκB
NFκB signaling pathway
NFκB
DNA
VEGF
Angiogenesis
VEGF signaling pathway
iNOS
MMP2/9
nNOS
PKCζ
c-Raf
MEK
ERK1/2
Relaxin
RXFP1
GαoB
Gαi3
GαS
Gβγ
PI3K
PIP3
AKT
eNOS
PI3K-Akt signaling pathway
NO
ET-1
ETB
Gαo
ET-1-32
bigET-1
(Vasodilator mechanisms)
MMP2
MMP9
MMP1
MMP13
Vasodilation
Vascular smooth muscle contraction
TGFβ
TGFβRI
TGFβRII
pSmad2
αSMA
Collagen
Anti-fibrosis
TGF-β signaling pathway
(Brain)
Increase in fluid intake
(Kidney)
Increase in effective renal plasma flow, GFR, and sodium excretion
Reduction in fibrosis
(Prostate)
Increases in sperm motility
(Ovary)
Follicle maturation
(Uterus)
Endometrial thickening
Angiogenesis
(Lungs)
Increases in lung perfusion and gas exchange
Relaxation of bronchi
Decrease in mast cell degradation and inflammatory leukocytes, and fibrosis
(Liver)
Decrease in fibrosis
(Vasculature)
Reduction in blood pressure
Vasodilation
(Heart)
Increase in atrial contraction and force
Decrease in fibrosis
RXFP2 Signaling
AC
Relaxin
INSL3
RXFP2
Gene transcription
(Testis, Ovary)
Germ cell maturation
(Gubernaculum)
Testicular descent
RXFP3 Signaling
Relaxin3
RXFP3
Gαi2
PLC
PKC
MKK4/7
p38
JNK
AP1
Ras
c-Raf
MEK
ERK1/2
MAPK signaling pathway
Src
Shc
SOS
Grb
β-arrestin
Internalisation
Transactivation
EGFR
(Brain)
Modulation of:
-responses to stress
-spatial and emotional memory
-feeding and metabolism
-motivation and reward
-circadian rhythm
-sleep/wake states
RXFP4 Signaling
INSL5
RXFP4
Gα16
IP3
DAG
Ca2+
Calcium signaling pathway
(Digestive system)
Appetite stimulation
Colon motility activation
Data on KEGG graph
Rendered by Pathview

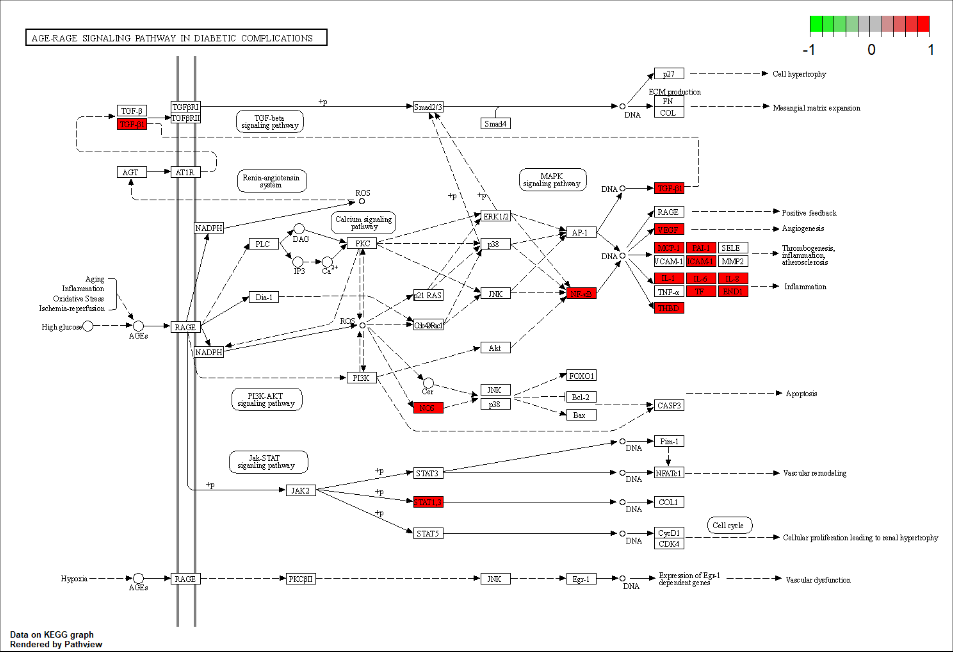
AGE-RAGE SIGNALING PATHWAY IN DIABETIC COMPLICATIONS
-1
0
1
TGF-β
TGF-β1
TGFβRI
TGFβRII
+p
Smad2/3
Smad4
TGF-beta signaling pathway
DNA
p27
Cell hypertrophy
ECM production
FN
COL
Mesangial matrix expansion
AGT
AT1R
Renin-angiotensin system
ROS
MAPK signaling pathway
DNA
TGF-β1
RAGE
Positive feedback
VEGF
Angiogenesis
MCP-1
PAI-1
SELE
VCAM-1
ICAM-1
MMP2
Thrombogenesis, inflammation, atherosclerosis
IL-1
IL-6
IL-8
TNF-α
TF
END1
Inflammation
THBD
NADPH
Calcium signaling pathway
ERK1/2
AP-1
PLC
DAG
PKC
p38
IP3
Ca2+
Aging
Inflammation
Oxidative Stress
Ischemia-reperfusion
Dia-1
p21 RAS
JNK
NF-κB
High glucose
AGEs
RAGE
ROS
Cdc42/Rac1
NADPH
Akt
PI3K
FOXO1
Cer
JNK
Bcl-2
p38
NOS
Bax
CASP3
Apoptosis
PI3K-AKT signaling pathway
Jak-STAT siganling pathway
Pim-1
STAT3
NFATc1
Vascular remodeling
JAK2
STAT1,3
COL1
Cell cycle
STAT5
CycD1
CDK4
Cellular proliferation leading to renal hypertrophy
Hypoxia
RAGE
PKCβII
JNK
Egr-1
Expression of Egr-1 dependent genes
Vascular dysfunction
Data on KEGG graph
Rendered by Pathview

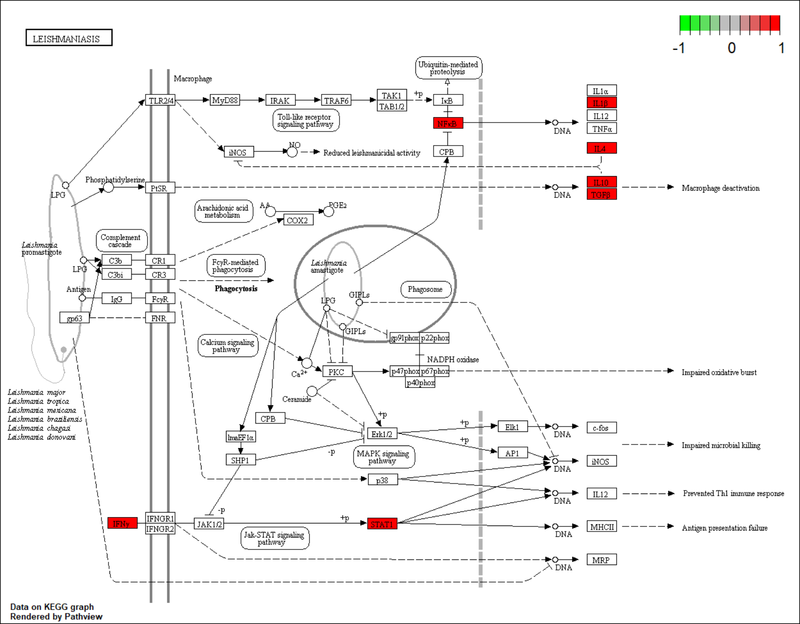
LEISHMANIASIS
-1
0
1
Macrophage
TLR2/4
MyD88
IRAK
TRAF6
TAK1
TAB1/2
+p
IκB
Ubiquitin-mediated proteolysis
Toll-like receptor signaling pathway
NFκB
CPB
IL1α
IL1β
IL12
TNFα
IL4
DNA
iNOS
NO
Reduced leishmanicidal activity
LPG
Phosphatidylserine
PtSR
IL10
TGFβ
Macrophage deactivation
Arachidonic acid metabolism
AA
COX2
PGE2
Complement cascade
Leishmania promastigote
C3b
C3bi
CR1
CR3
FcγR-mediated phagocytosis
Phagocytosis
Antigen
IgG
FcγR
gp63
FNR
Leishmania amastigote
Phagosome
GIPLs
gp91phox
p22phox
NADPH oxidase
Calcium signaling pathway
Ca2+
PKC
p47phox
p67phox
p40phox
Impaired oxidative burst
Ceramide
CPB
lmaEF1α
SHP1
Erk1/2
MAPK signaling pathway
-p
Elk1
AP1
c-fos
Impaired microbial killing
p38
Prevented Th1 immune response
IFNγ
IFNGR1
IFNGR2
JAK1/2
Jak-STAT signaling pathway
STAT1
MHCII
Antigen presentation failure
MRP
Leishmania major
Leishmania tropica
Leishmania mexicana
Leishmania braziliensis
Leishmania chagasi
Leishmania donovani
Data on KEGG graph
Rendered by Pathview

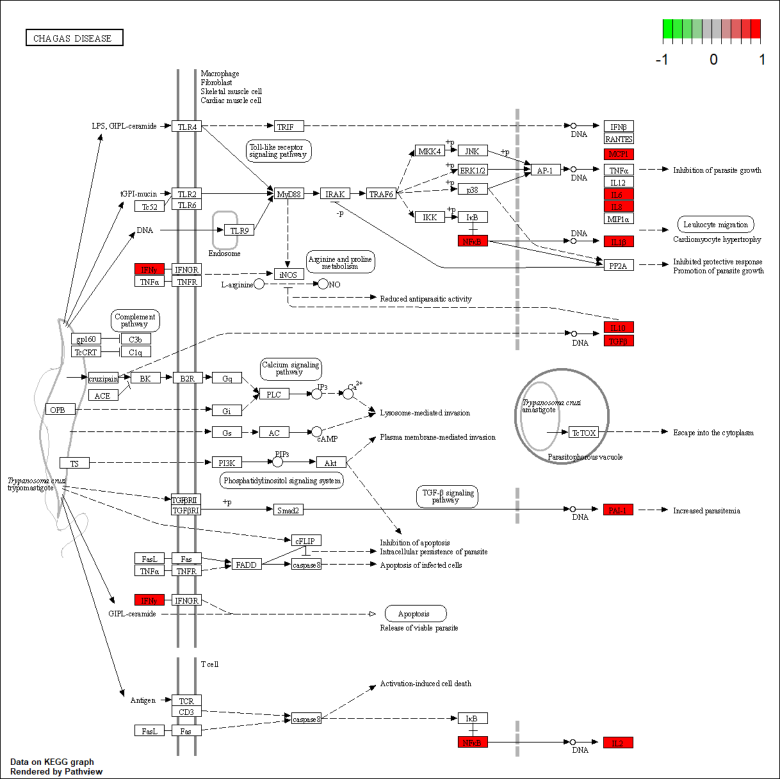

CHAGAS DISEASE
-1
0
1
Macrophage
Fibroblast
Skeletal muscle cell
Cardiac muscle cell
LPS, GIPL-ceramide
TLR4
TRIF
IFNβ
RANTES
MCP1
Toll-like receptor signaling pathway
MKK4
JNK
ERK1/2
p38
AP-1
DNA
TNFα
IL12
IL6
IL8
MIP1α
Inhibition of parasite growth
tGPI-mucin
Tc52
TLR2
TLR6
MyD88
IRAK
TRAF6
IKK
IκB
NFκB
IL1β
Leukocyte migration
Cardiomyocyte hypertrophy
PP2A
Inhibited protective response
Promotion of parasite growth
TLR9
Endosome
IFNγ
IFNGR
TNFR
iNOS
Arginine and proline metabolism
L-arginine
NO
Reduced antiparasitic activity
Complement pathway
gp160
C3b
TcCRT
C1q
IL10
TGFβ
cruzipain
BK
B2R
Gq
ACE
Calcium signaling pathway
PLC
IP3
Ca2+
Lysosome-mediated invasion
OPB
Gi
Gs
AC
cAMP
Plasma membrane-mediated invasion
TS
PI3K
PIP3
Akt
Phosphatidylinositol signaling system
Trypanosoma cruzi amastigote
TcTOX
Escape into the cytoplasm
Parasitophorous vacuole
Trypanosoma cruzi trypomastigote
TGFβRII
TGFβRI
Smad2
TGF-β signaling pathway
PAI-1
Increased parasitemia
cFLIP
Inhibition of apoptosis
Intracellular persistence of parasite
FasL
Fas
FADD
caspase8
Apoptosis of infected cells
GIPL-ceramide
Apoptosis
Release of viable parasite
T cell
Activation-induced cell death
Antigen
TCR
CD3
IL2
Data on KEGG graph
Rendered by Pathview

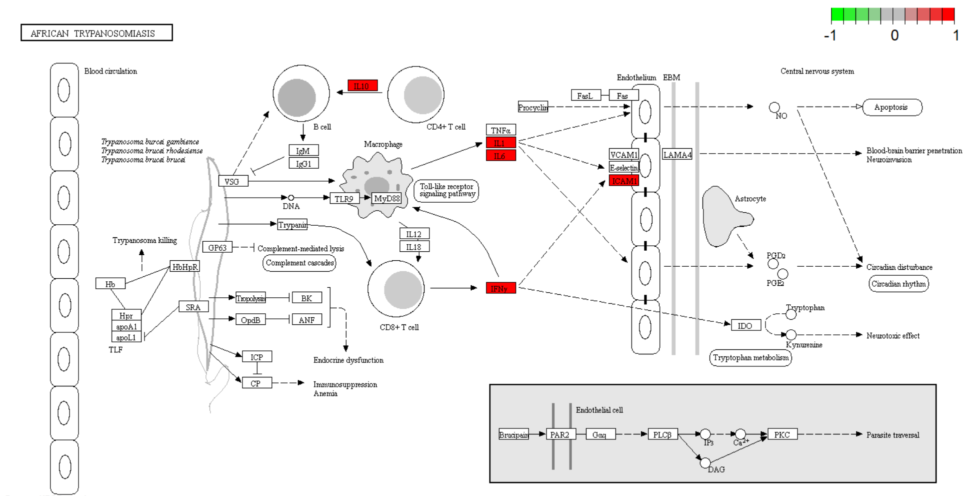

Data on KEGG graph
Rendered by Pathview

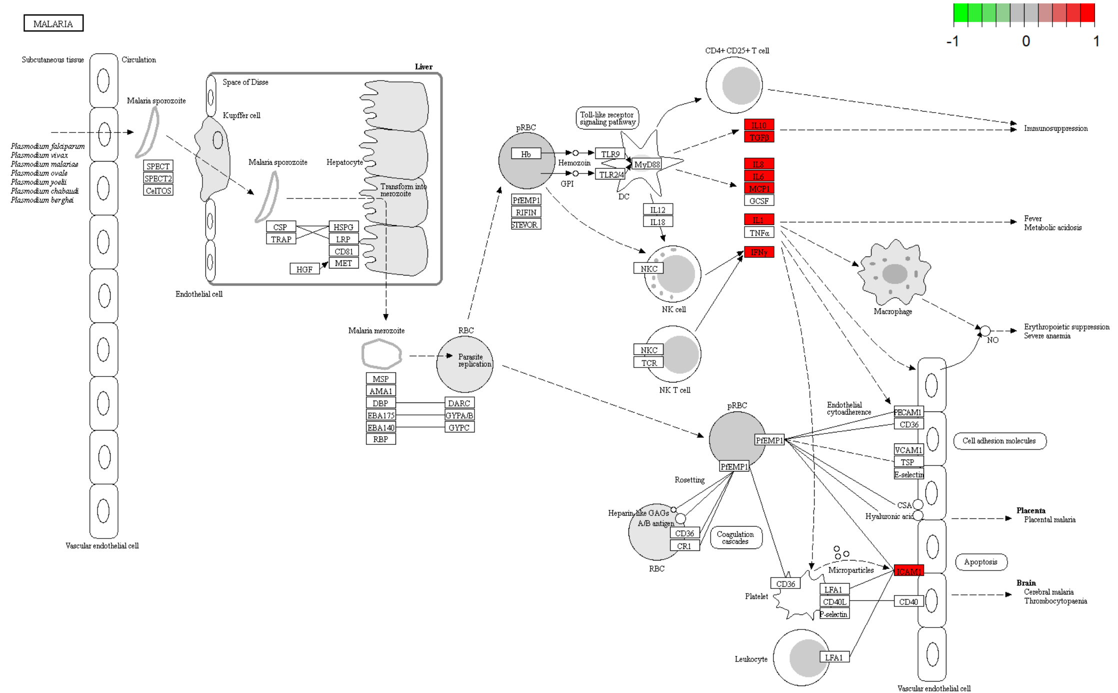

Data on KEGG graph
Rendered by Pathview

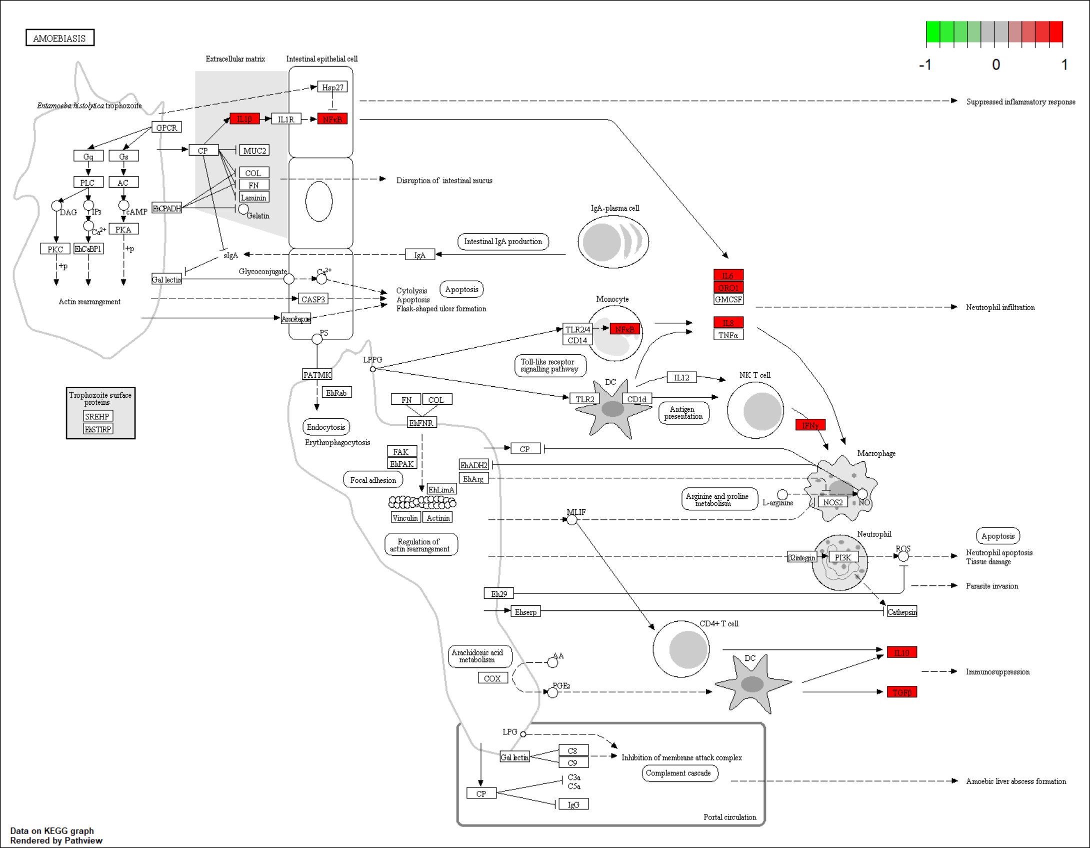
AMOEBIASIS
-1
0
1
Extracellular matrix
Intestinal epithelial cell
Entamoeba histolytica trophozoite
Hsp27
IL1β
IL1R
NFκB
Suppressed inflammatory response
GPCR
Gq
Gs
PLC
AC
DAG
IP3
cAMP
Ca2+
PKA
PKC
EhCaBP1
+p
Actin rearrangement
CP
MUC2
COL
FN
Laminin
Gelatin
EhCPADH
Disruption of intestinal mucus
IgA-plasma cell
Intestinal IgA production
sIgA
IgA
Gal lectin
Glycoconjugate
CASP3
Amoebapore
Cytolysis
Apoptosis
Flask-shaped ulcer formation
PS
IL6
GRO1
GMCSF
Neutrophil infiltration
IL8
TNFα
Monocyte
TLR2/4
CD14
Toll-like receptor signalling pathway
LPPG
PATMK
EhRab
Endocytosis
Erythrophagocytosis
Trophozoite surface proteins
SREHP
EhSTIRP
DC
TLR2
CD1d
IL12
NK T cell
Antigen presentation
IFNγ
FN
COL
EhFNR
FAK
EhPAK
Focal adhesion
EhLimA
Vinculin
Actinin
Regulation of actin rearrangement
EhADH2
EhArg
Macrophage
Arginine and proline metabolism
L-arginine
NOS2
NO
MLIF
Neutrophil
β2integrin
PI3K
ROS
Apoptosis
Neutrophil apoptosis
Tissue damage
Parasite invasion
Eh29
Ehserp
Cathepsin
CD4+ T cell
IL10
Immunosuppression
TGFβ
Arachidonic acid metabolism
AA
COX
PGE2
LPG
Gal lectin
C8
C9
Inhibition of membrane attack complex
Complement cascade
Amoebic liver abscess formation
C3a
C5a
IgG
Portal circulation
Data on KEGG graph
Rendered by Pathview

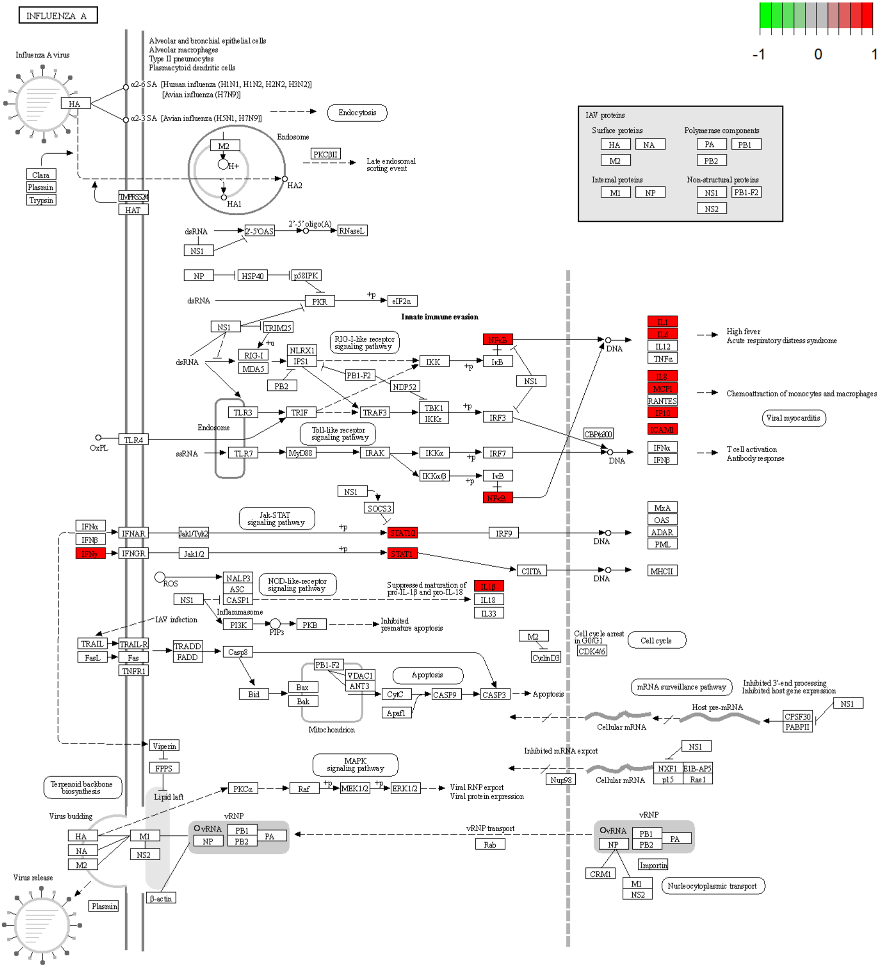

Data on KEGG graph
Rendered by Pathview

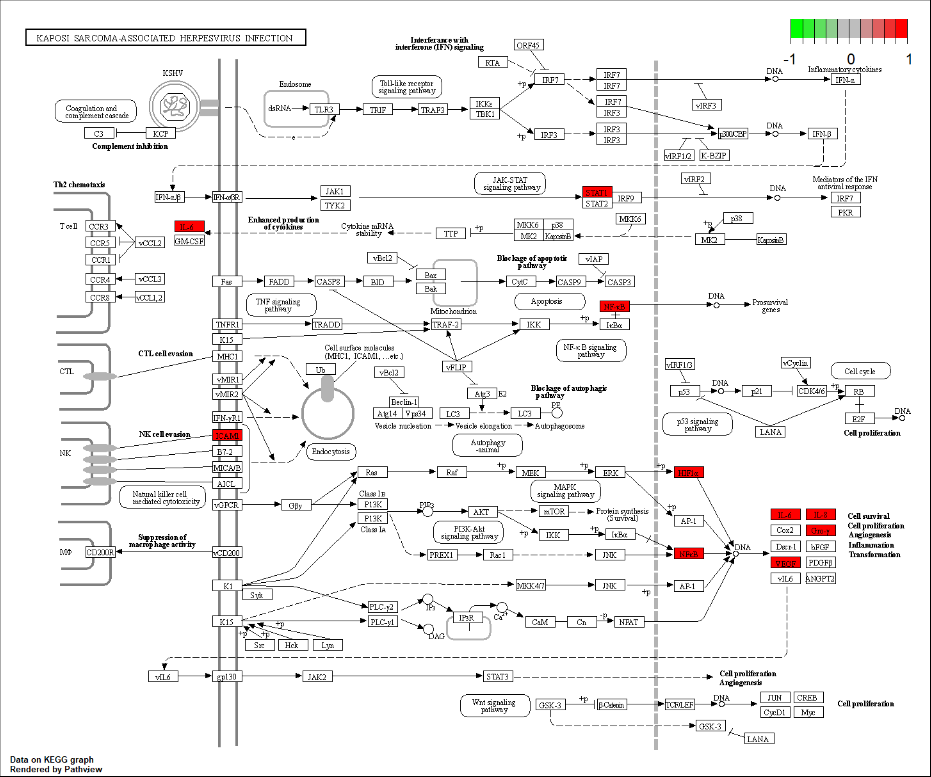
KAPOSI SARCOMA-ASSOCIATED HERPESVIRUS INFECTION
-1
0
1
KSHV
Coagulation and complement cascade
C3
KCP
Complement inhibition
Endosome
dsRNA
TLR3
TRIF
TRAF3
Toll-like receptor signaling pathway
Interferance with interferone (IFN) signaling
ORF45
RTA
IKKε
TBK1
IRF7
IRF3
+p
vIRF3
p300/CBP
vIRF1/2
K-BZIP
DNA
Inflammatory cytokines
IFN-α
IFN-β
Th2 chemotaxis
T cell
CCR3
CCR5
CCR1
CCR4
CCR8
vCCL2
vCCL3
vCCL1,2
IFN-α/β
IFN-α/βR
JAK1
TYK2
JAK-STAT signaling pathway
STAT1
STAT2
IRF9
vIRF2
Mediators of the IFN antiviral response
PKR
IL-6
GM-CSF
Enhanced production of cytokines
Cytokine mRNA stability
TTP
MKK6
p38
MK2
KaposinB
vBcl2
Bax
Bak
Mitochondrion
Blockage of apoptotic pathway
vIAP
Fas
FADD
CASP8
BID
CytC
CASP9
CASP3
Apoptosis
TNF signaling pathway
NF-κB
Prosurvival genes
TNFR1
TRADD
TRAF-2
IKK
IκBα
K15
CTL
CTL cell evasion
MHC1
Cell surface molecules (MHC1, ICAM1, ...etc.)
Ub
vFLIP
NF-κB signaling pathway
vMIR1
vMIR2
Beclin-1
Atg14
Vps34
Atg3
E2
Blockage of autophagic pathway
LC3
PE
Vesicle nucleation
Vesicle elongation
Autophagosome
Autophagy - animal
vIRF1/3
vCyclin
Cell cycle
p53
p21
CDK4/6
RB
E2F
LANA
p53 signaling pathway
Cell proliferation
IFN-γR1
NK
NK cell evasion
ICAM1
B7-2
MICA/B
AICL
Endocytosis
Natural killer cell mediated cytotoxicity
vGPCR
Gβγ
Ras
Raf
MEK
ERK
HIF1α
MAPK signaling pathway
Class IB
P13K
Class IA
PIP3
AKT
mTOR
Protein synthesis (Survival)
PI3K-Akt signaling pathway
IκBα
AP-1
NFκB
IL-8
Cox2
Gro-γ
Dscr-1
bFGF
VEGF
PDGFβ
vIL6
ANGPT2
Cell survival
Cell proliferation
Angiogenesis
Inflammation
Transformation
MΦ
CD200R
Suppression of macrophage activity
vCD200
PREX1
Rac1
JNK
K1
Syk
MKK4/7
PLC-γ2
PLC-γ1
IP3
IP3R
Ca2+
CaM
Cn
NFAT
DAG
Src
Hck
Lyn
vIL6
gp130
JAK2
STAT3
Cell proliferation
Angiogenesis
Wnt signaling pathway
GSK-3
β-Catenin
TCF/LEF
JUN
CREB
CycD1
Myc
Data on KEGG graph
Rendered by Pathview

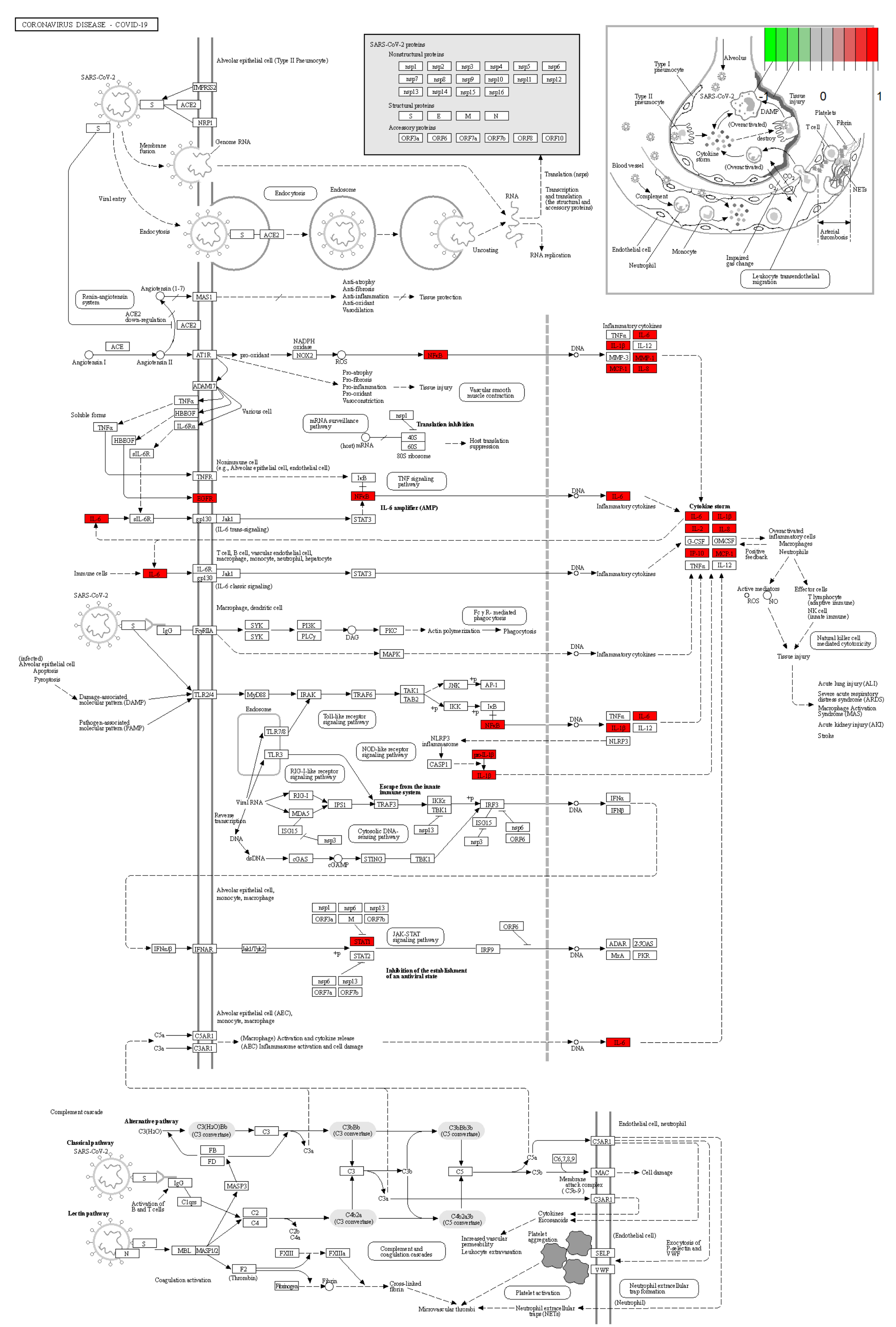

Data on KEGG graph
Rendered by Pathview

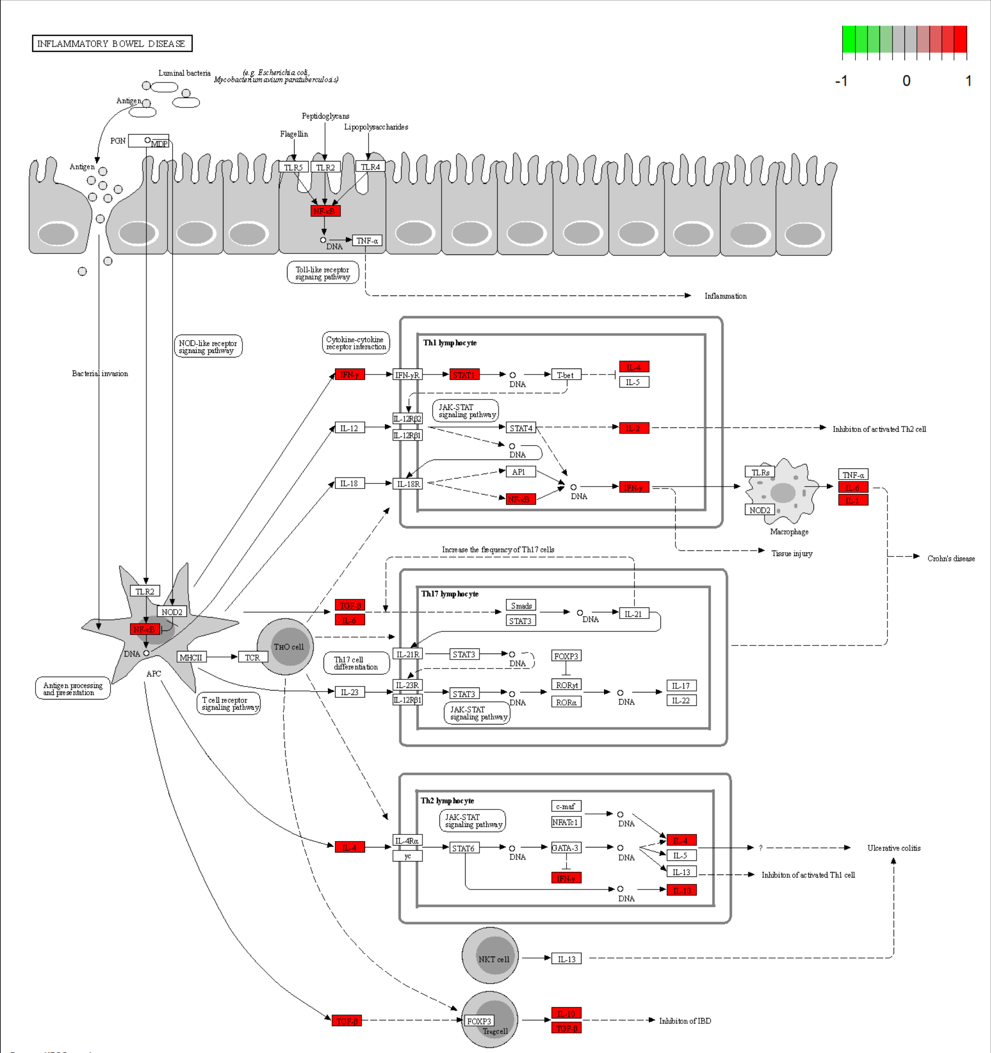

Data on KEGG graph
Rendered by Pathview

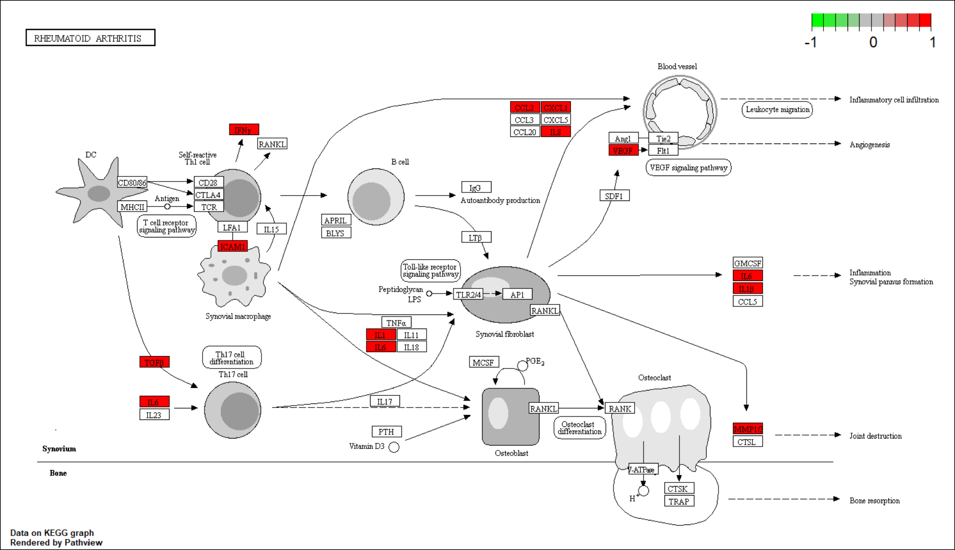
RHEUMATOID ARTHRITIS
-1
0
1
DC
CD80/86
MHCII
Antigen
Self-reactive Th1 cell
CD28
CTLA4
TCR
T cell receptor signaling pathway
IFNγ
RANKL
LFA1
ICAM1
IL15
Synovial macrophage
B cell
APRIL
BLYS
IgG
Autoantibody production
LTβ
Toll-like receptor signaling pathway
Peptidoglycan
LPS
TLR2/4
AP1
RANKL
Synovial fibroblast
TNFα
IL1
IL11
IL6
IL18
CCL2
CXCL1
CCL3
CXCL5
CCL20
IL8
Blood vessel
Leukocyte migration
Inflammatory cell infiltration
Ang1
Tie2
VEGF
Flt1
VEGF signaling pathway
Angiogenesis
SDF1
GMCSF
IL6
IL1β
CCL5
Inflammation
Synovial pannus formation
TGFβ
Th17 cell differentiation
Th17 cell
IL6
IL23
IL17
MCSF
PGE2
PTH
Vitamin D3
RANKL
Osteoblast
Osteoclast differentiation
Osteoclast
RANK
MMP1/3
CTSL
Joint destruction
V-ATPase
H+
CTSK
TRAP
Bone resorption
Synovium
Bone
Data on KEGG graph
Rendered by Pathview

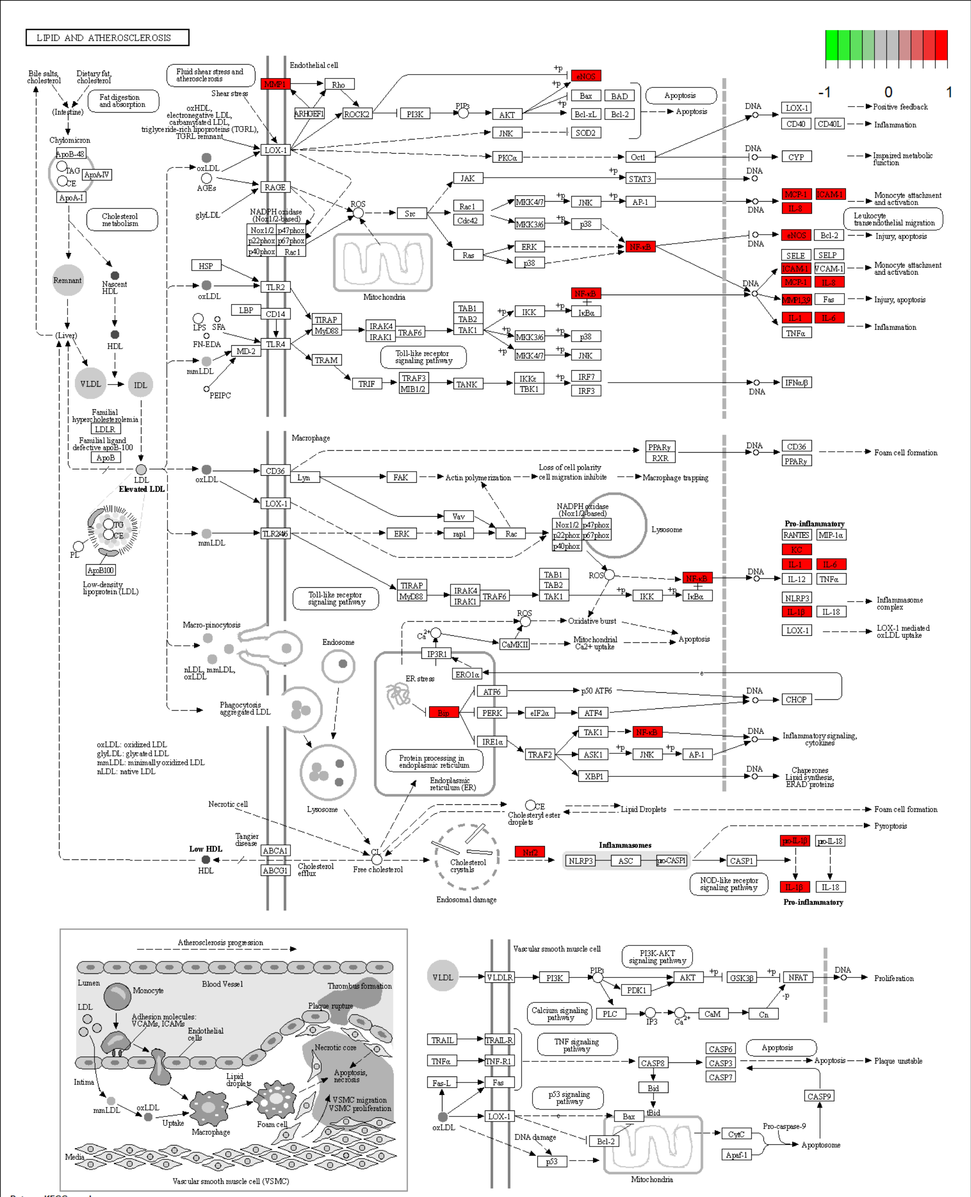

Data on KEGG graph
Rendered by Pathview

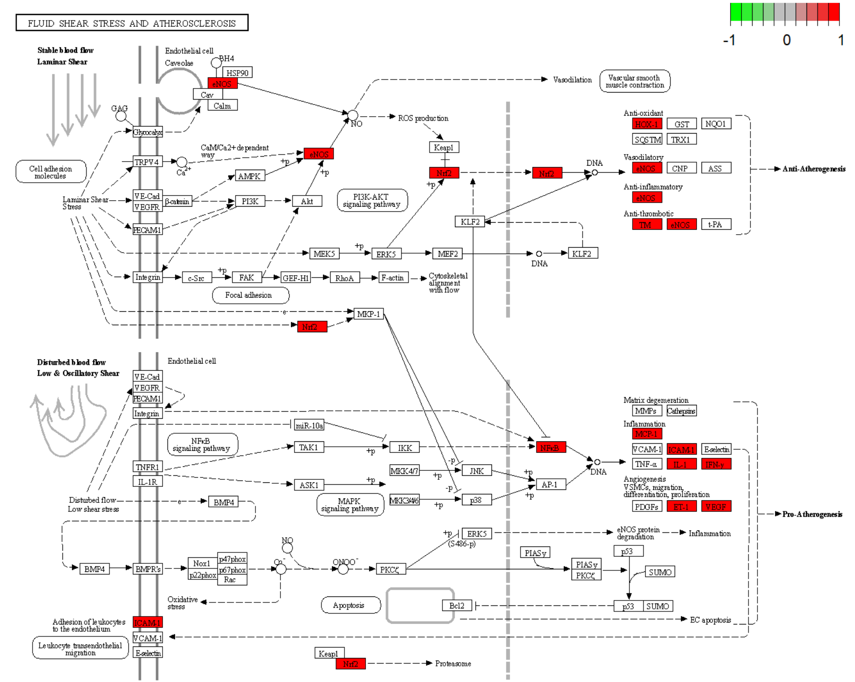

Data on KEGG graph
Rendered by Pathview
